# Multi-domain feature fusion with variational mode decomposition and hybrid LightGBM-Logistic Regression for multi-class seizure classification

**DOI:** 10.1186/s13040-026-00578-6

**Published:** 2026-06-22

**Authors:** A. Aameer Arshath, D. Karthikeyan

**Affiliations:** Vellore Institute of Technology, School of Computer Science Engineering and Information Systems, Vellore, India

**Keywords:** Class imbalance, EEG preprocessing, Feature selection, Hyperparameter tuning, Signal denoising, SMOTE

## Abstract

**Objective:**

The problem of seizure classification using multi-seizure types based on reduced-channel EEG recordings is difficult due to the complexity and nonlinearity of seizure patterns and their high class imbalance.

**Methods:**

In this paper, a reduced-channel EEG-based method is proposed to perform automated multi-seizure classification using the Temple University Seizure Corpus (TUSZ) data set. The proposed approach uses the T3 and T4 EEG temporal channels and performs Variational Mode Decomposition (VMD) to decompose EEG signals into several intrinsic mode functions to extract the relevant temporal and spectral features of seizures. Next, multi-domain features, including statistical, spectral, entropy, and nonlinear features, are extracted from each VMD mode. To avoid the redundancy of the extracted features and preserve seizure-related features, an MI-RFE feature selection scheme is used. The extracted features were then classified using a stacked ensemble model incorporating LightGBM and logistic regression. For the purpose of ensuring unbiased testing and avoiding any information leakage, the Fold-Wise SMOTE technique was used in the process of stratified K-fold cross-validation.

**Results:**

In experimental analysis of the developed technique with nine types of seizures, positive outcomes were obtained in terms of classification accuracy that included high classification accuracy, high weighted F1 measure, and multiclass ROC-AUC characteristics, resulting in classification accuracy of 92.16%. Also, when comparing the developed feature selection approach with standard techniques, the approach’s efficiency was demonstrated.

**Significance:**

The attained results show that the suggested approach for reducing the number of channels used in analyzing brain dynamics is effective.

**Clinical trial number:**

Not applicable.

## Introduction

Seizures are sudden events of unusual electrical activity in the brain that can have severe impacts on motor function, awareness, and quality of life. Proper identification of seizure types is important for informing therapeutic approaches and outcomes. Electroencephalograms (EEGs) of the brain are the standard for measuring brain activity with high temporal resolution [1]. EEGs are particularly attractive for seizure detection because they are non-invasive, inexpensive, and portable. Nonetheless, developing seizure diagnoses from EEGs is an inherently difficult problem, as the signals are extremely non-linear, non-stationary, and will often also be contaminated by muscle activity and other movement artifacts. Moreover, waveform differences (that are often very subtle) may be responsible for differentiating seizure types, which further add to the complexity of a neurologist’s interpretation [2, 3]. Thus, the complexity surrounding the incident and diagnosis of a seizure has inspired researchers to develop automated frameworks for seizure classification based upon enhanced signal processing and ML techniques to detect and classify seizures with high accuracy and speed [4, 5].

Traditional studies have extracted meaningful patterns from the EEG signals using a variety of decomposition and signal representation techniques, including wavelet transforms [13], empirical mode decomposition [14], and short-time Fourier transforms [15]. Most recent studies have adopted DL architectures, including CNNs and RNNs, to capture complex temporal–spectral dependencies [8, 16]. While these studies have shown promising results, several studies have investigated only specific feature types, accounted for the variability between classes poorly, or were limited by the use of unbalanced datasets [12].

Current advances in seizure classification via EEG data have seen considerable interest in the areas of hybrid deep learning, the combination of multi-channel EEG signals, and intelligent feature selection to improve the multiclass seizure classification task. For instance [17], employed a hybrid deep learning algorithm, which uses Hjorth features based on time and time-frequency analysis in their epileptic seizure classification problem. Meanwhile [18], came up with a new deep wavelet spectrum network combining multi-channel EEG signals for epilepsy EEG signal classification. Furthermore [19], designed a real-time deep learning algorithm to classify multi-seizures from EEG signals for children. In addition [20], proposed a hybrid artificial intelligence (AI)-based EEG/non-EEG algorithm to predict epileptic seizures. Besides [21], applied an optimized feedforward neural network to automatically detect seizures, while [22] developed AI-driven algorithms for electrographic seizure classification and seizure onset detection.

To fill these gaps, we propose a VMD-based, multi-domain feature fusion approach with the following attributes: (1) explores the temporal channels (T3, T4) in depth, (2) integrates time, frequency, and time-frequency domain features, (3) employs multiple feature selection methods [23–25], and (4) integrates a hybrid LightGBM - logistic regression classifier. Together, these attributes will enhance the accuracy, interpretability, and generalizability of the multi-class seizure classification supported by mode-wise and class-wise performance results.

### Limitations and challenges in EEG-based seizure classification

Although substantial development has been made in epileptic seizure classification using EEG, there still exist several dilemmas that hinder the accuracy and generalization and clinical feasibility of the established approaches. An important issue is the strong inconsistency among datasets and patient populations. For example, the TUSZ dataset is comprehensive, despite high class imbalance and seizure type variability that makes multi-class classification difficult Table 1 [6]. Correspondingly, datasets focusing on pediatrics, such as CHB-MIT, suffer from both inter-subject variability and generalization problems, as models trained on one patient group will rarely work well on new patients [7]. Datasets like Bonn University’s are widely used for benchmarking and achieve high accuracy (98%+ with Bi-LSTM [8]), but the range is restricted to single-channel artifact-free EEG, which is impractical for clinical multi-seizure activity. These data-related issues illustrate the delicate balance between benchmarking performance and clinical and real-world application to classify seizure types. The second barrier depends on how feature extraction and calculations and tests are created.Traditional wavelet transform or EMD-based methods have demonstrated effectiveness but cannot capture the full complexity of seizure dynamics. Recent works have investigated CNN-LSTM hybrids using time–frequency representations Table 2 [2, 26], and models such as Bi-LSTM [29] with attention networks.

**Table 1:** Overview of machine learning approaches for EEG-based seizure classification in existing literature

Dataset	Type	Scope	Notable Results	Challenges	References
TUSZ	Scalp EEG	Multi-seizure classification	Accuracy: 92.00% (CE-stSENet)	Class imbalance, variability in seizure types	[6]
CHB-MIT	Scalp EEG	Pediatric, multi-channel	Accuracy: 97.09% (LightSeizureNet), 95.38% (Geometric Deep Learning)	Inter-subject variability, generalizability issues	[7]
Bonn University	Single-channel EEG	Binary and multi-class classification	Accuracy: 98%+ (Path Signature and Bi-LSTM)	Limited to single-channel recordings, not suitable for multi-seizure types	[8]
SWEC-ETHZ	Intracranial EEG (iEEG)	Seizure onset zone (SOZ) analysis	Sensitivity: 97.5%, FDR: 0.06/hour (Transformer-based model)	High memory requirements, limited generalization	[9]
TJU-HH	Intracranial EEG (iEEG)	Continuous seizure detection	Latency: 9.9 seconds, sensitivity: 98.1%	Patient-independent algorithms underexplored	[9]
EPILEPSIAE	Multi-modal (EEG + ECG)	Complex partial, simple partial, generalized	Sensitivity: 90.24%, Specificity: 91.58% (Federated Learning)	Computational demands, dependency on distributed resources	[10]
Private Datasets	Varies	Patient-specific seizure detection	Accuracy: 99.40% (Path Signature and Bi-LSTM), strong results in cross-patient experiments	Limited accessibility, lack of standardization	[8]
Siena EEG	Scalp EEG	Subject-independent prediction	Sensitivity: 97.10%	Dataset-specific biases, scalability issues	[11]
Bonn University	Single-channel EEG	Foundational binary classification	Accuracy: 94.60% (multi-class tasks)	Artifact-free dataset, not representative of real-world scenarios	[12]

TUSZ – Temple University Hospital Seizure Dataset;CHB-MIT – Children’s Hospital Boston–Massachusetts Institute of Technology EEG Dataset;SWEC–ETHZ – Swiss Epilepsy Center–Swiss Federal Institute of Technology Zurich Dataset;TJU–HH – Tianjin University–Henan Hospital Intracranial EEG Dataset;EPILEPSIAE – European Epilepsy Database;CNN – Convolutional Neural Network;CNN–LSTM – Convolutional Neural Network–Long Short–Term Memory Network;1D–CNN – One–Dimensional Convolutional Neural Network;Bi–LSTM – Bidirectional Long Short–Term Memory Network;CE–stSENet – Channel–Enhanced Spatial–Temporal Squeeze–and–Excitation Network; FDR – False Detection Rate

**Table 2:** Methods for categorizing focal, generalized, and non-seizure events utilizing scalp EEG in the TUSZ dataset

Publications	Features	Classifier	Performance
[6]	Spectral and temporal features	CE-stSENet	Accuracy: 92.00%
[26]	HVD, Time-frequency images	CNN-LSTM	Accuracy: 98.82%, Weighted F1-Score: 98.80%
[2]	Time-frequency features	CNN-LSTM	Accuracy: 98.82%
[12]	Time and frequency domain features	1D-CNN	Accuracy: 99.00%
[27]	Data augmentation	CNN	Accuracy: 92.00%
[8]	Path signature features, Attention mechanism	Bi-LSTM	Accuracy: 99.40%
[11]	EEG adjacency matrices	Graph Neural Network	Accuracy: 96.05%, Sensitivity: 97.10%, Specificity: 95.60%
[28]	Time and frequency domain features	Multi-View CNN	AUC: 0.92, Latency: 1 second
[10]	EEG and ECG signals	Federated Learning (Res1DCNN)	Sensitivity: 90.24%, Specificity: 91.58%

HVD—Hilbert Vibration Decomposition;GNN—Graph Neural Network AUC—Area Under the Receiver Operating Characteristic Curve

However, such strategies tend to be computationally intensive, dataset-dependent, and lack transparency, rendering them unfit for resource-limited or clinical settings. Additionally, scalp EEG-trained models like CE-stSENet with 92% accuracy on TUSZ Table 2 continue to suffer from imbalanced class distributions as well as the inability to identify subtle seizure type variations. Other methods, including federated learning on EPILEPSIAE, are promising for multi-modal integration Table 1, but come with high computational demands and reliance on distributed resources, making them less scalable.

In summary, EEG-based seizure classification challenges fall into the following categories: **(i) issues related to datasets** like imbalance, variability, and limited access (TUSZ [6], CHB-MIT [7], Bonn [8], Siena EEG [11]); **(ii) feature extraction** limitations where handcrafted or domain-specific features do not generalize across heterogeneous seizure types (1D-CNN [12], CNN-LSTM [26]); and **(iii) model limitations** where state-of-the-art architectures are computationally expensive but provide high accuracy and lack interpretability or perform poorly across patients (Federated Learning [10], GNN [30]). Solving these problems necessitates strong decomposition techniques, multi-domain feature fusion [31], balanced learning approaches, and hybrid models that can balance accuracy, interpretability, and efficiency—directions that inspire the proposed framework in this work.

### Existing gaps and study motivation

The literature on automatic EEG-based seizure classification is rapidly developing and clearly demonstrates the power of ML and DL to support the clinical diagnosis of epilepsy.First, there are limitations in datasets, including class imbalance, issues with inter-subject variability (note that collecting EEG data does require medical ethics approval), and limitations in generalizability (TUSZ [6], CHB-MIT [7], Bonn [8]). Numerous high-performing approaches show accuracies above 95% on benchmark datasets, yet we know these types of models drop in performance for unseen patients or more broadly defined clinical data. Second, many feature extraction methods limit themselves to single analysis domains such as spectral (or temporal) features, without considering complementary information in frequency bands or time-frequency representations. Producing features specific to a single domain limits the chance of fully capturing the proliferation of complex, non-linear, and potentially non-stationary properties of seizure signals, which should be better represented with analysis across multiple domains. Third, there are approaches that demonstrate impressive performance, such as CNN-LSTM, Bi-LSTM with attention, and GNNs Table 2 [11], but they either suffer from computational overhead, decreased interpretability, or reliance on dataset-specific biases, which render them impractical for clinical use.

These issue open the possibility of creating a framework that establishes a proper balance between accuracy, interpretability, and efficiency while overcoming challenges required to address dataset and inter-class imbalance. With these gaps in mind, I have developed a VMD (variational mode decomposition)-based multi-domain feature fusion pipeline that focuses on the seizure-prone temporal channels T3 and T4. Employing VMD to decompose EEG into five intrinsic modes, extracting all the three domain features, and then using multiple feature selection methods (SelectKBest MI-RFE,SelectKBest,Light GBM based, and RFE) allows us to build a rich and discriminative feature space. Classification is conducted using a hybrid LightGBM-Logistic Regression model, which collectively exploits the generalization ability of a type of gradient boosting and interpretability of linear models.

Our research addresses directly three key gaps in the existing literature:

- **Multi-domain integration:** overcomes the limited analysis of features through a single-domain approach.
- **Balanced and discriminative feature selection:** recognizes the non-linearity, redundancy, and the imbalanced nature of seizure datasets.
- **Hybrid and interpretable modeling:**achieves the best predictive performance while providing substantial clinical relevance and feasibility.

Thus, the work proposed in this research not only advances the state of the art but also lays the groundwork for scalable and computationally efficient seizure classification framework.

### Proposed approach and contributions

This study has proposed an accurate and interpretable multiclass seizure classification system using a VMD-driven multi-domain feature fusion framework. Although individual components such as Variational Mode Decomposition (VMD), feature extraction techniques, and ensemble classifiers have been previously investigated in EEG analysis, the novelty of the proposed work lies in the integration of these components within a unified reduced-channel multi-seizure classification framework. Unlike many existing studies that rely on high-density multichannel EEG recordings or binary seizure detection, the proposed methodology focuses on nine-class seizure classification using only T3 and T4 temporal EEG channels. In the complete workflow, EEG recording is derived from the TUSZ v2.0.3 dataset comprising scalp recordings located on T3 and T4 temporal channels. Only the T3 and T4 temporal EEG channels from the TUSZ dataset were considered in this work for seizure analysis. This selection was made since the temporal areas have been shown to be strongly linked to epileptiform abnormalities and have a high likelihood of significant rhythmic spike discharges during focal and temporal lobe seizures. Moreover, reduced-channel EEG systems are important to design lightweight, efficient, and wearable seizure monitoring devices. Although generalized and extra-temporal seizure dynamics may involve wider cortices, there is evidence that seizures could propagate to the temporal electrodes by means of network synchronization phenomena. As such, the proposed framework evaluates whether discriminative information contained in the T3/T4 channels alone is enough for discriminating multiple types of seizures in a reduced-channel scenario. It must be recognized, however, that a reduced number of two temporal electrodes might not sufficiently represent the full spatial dynamics of different kinds of seizures, especially generalized and extra-temporal focal seizures. Consequently, the proposed framework could be considered as a reduced-channel classification scheme rather than a substitute for comprehensive multichannel analysis of clinical EEG data. These central temporal channels were chosen to have clinical relevance in terms of determining seizure localization. The extracted EEG signals were transferred to functional EEG formats and decompose into five modes of intrinsic mode functions (Modes 1–5) using Variational Mode Decomposition (VMD). Compared to classical decomposition methods like wavelet transform and EMD, VMD provides a more stable and robust approach to separate discrete frequency bands–this stability is essential to reflect the dynamics of seizure.

From each mode we derive a diverse collection of features in three interrelated domains, the time, frequency, and time-frequency domains. In the time domain, we extract statistical and nonlinear features to provide an indication of the waveform shape and complexity index, features such as mean, variance, skewness, kurtosis, entropy, and Hjorth parameters. Features in the frequency domain are derived from spectral features such as power spectral density (PSD), mapping each mode to closely related EEG bands (delta, theta, alpha, beta, and gamma). We also provide time-frequency domain features, such as RMS energy, Teager-Kaiser energy, and spectral entropy, to define locality in capturing the temporal processes and spectral irregularities. The feature vectors are fused into a single representation, resulting in a rich feature space where there may be both multi-domain features derived from the three domains. Now from these each five modes, we are selecting the one mode, which has the highest value of frequency value (Fig. 12) compared to all the other four modes. From the selected mode, we can get only the mode with the maximum value of frequency in order to remove the unwanted features. Thus, the fused features include only those that are relevant.

To promote a compact and discriminative fused feature space, a variety of feature selection strategies were investigated. The first feature selection strategy was SelectKBest MI-RFE, which uses mutual information or mutual information for multivariate datasets, as this method is most appropriate for nonlinear and multi-class EEG data. As further validation of robustness and as a baseline to compare against performance,SelectKBest MI-based feature selection, LightGBM-based ranking feature selection, and Recursive Feature Elimination (RFE) were also applied. All of these selections avoid redundancy, alleviate the curse of dimensionality, and identify the seizure feature attributes that matter most. In conclusion, the SMOTE was employed during data pre-processing to address the imbalanced class distribution in TUSZ so that the seizure classes were more representatively balanced.

The hybrid LightGBM–Logistic Regression model is applied to classify the refined feature set. While LightGBM employs efficient gradient boosting to learn complex nonlinear decision boundaries, logistic regression offers interpretability and regularization. Thus, this hybrid design achieves a balance between interpretability, predictive ability, and robustness. The framework will characterize overall and classification performance (accuracy, precision, recall, F1-score, and kappa statistic), component-wise performance (i.e., contribution of each decomposed mode), and class-wise accuracy across 9 seizure types.

Key Contributions of this Work:

- Creating a unique, channel-based, VMD-driven decomposition pipeline applied to temporal EEG channels (T3 and T4) for increased biological relevance in seizure diagnosis.
- Applying a multi-domain feature fusion method using time, frequency, and time-frequency features in order to encapsulate the holistic characteristics of seizures.
- Evaluating multiple feature selection methods (SelectKBest MI-RFE, SelectKBest MI, LightGBM-based ranking method, and RFE) to ensure robustness in discriminative attribute selections.
- Using SMOTE class balancing methods to address seizure type imbalance in the TUSZ dataset.
- Creating a novel LightGBM-Logistic Regression hybrid classifier achieving competitive accuracy (95.16%) whilst maintaining interpretable outcomes.
- Modelling a full mode-wise and class-wise performance analysis for a better understanding of frequency-specific contributions and classifier generalization for the nine different seizure types available.

The remaining sections of the paper are laid out as follows. Section “Clinical background on seizures” provides details on the classes of seizures and the dataset. Section “Materials and methods” outlines the proposed VMD-based multi-domain classification framework, Section “Results and discussions” gives the implementations and performance analysis, and Section “Conclusion” concludes with main findings and future work directions.

## Clinical background on seizures

**Fig. 1:**
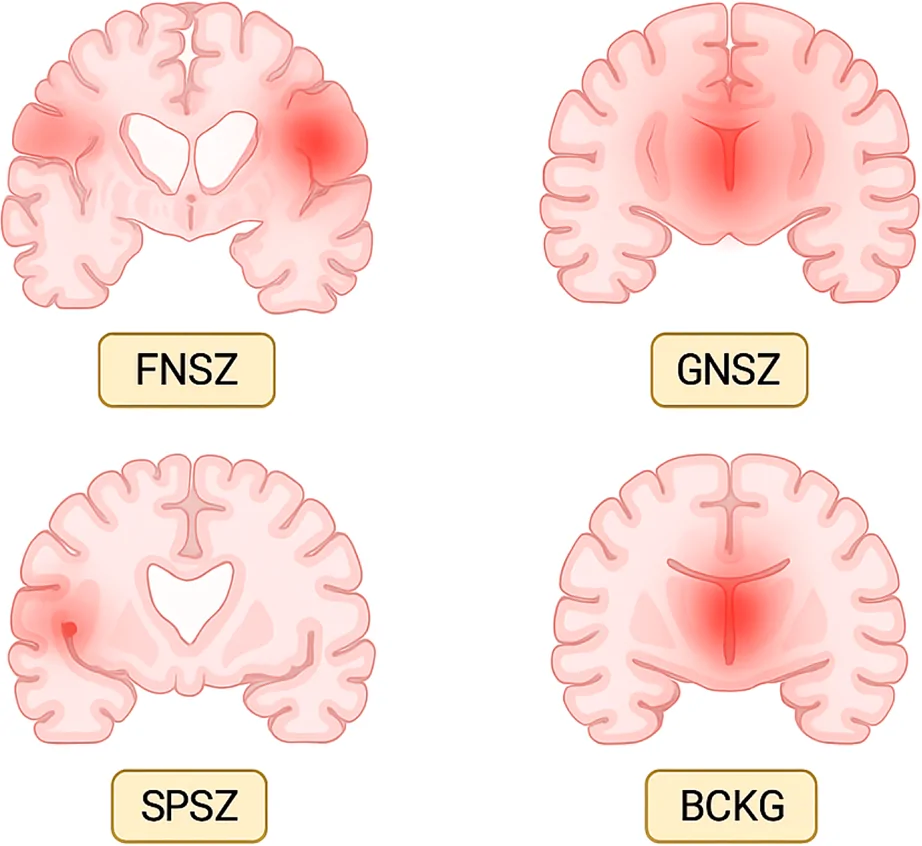
ILAE-based seizure type classification

Seizures are unexpected and unregulated electrical discharges in the brain that may show many different clinical presentations and patterns on the electroencephalography. Seizures can affect motor, sensory, cognitive, and behavioral functions based on their origin and spread. The ILAE provides a common and standardized approach to the classification of seizures into focal, generalized, and unknown onset. Figure 1 illustrates the seizure nine classes. This classification has been accepted and adopted into clinical practice worldwide and is critical in informing treatment strategies and improving diagnostic specificity.

**Fig. 2:**
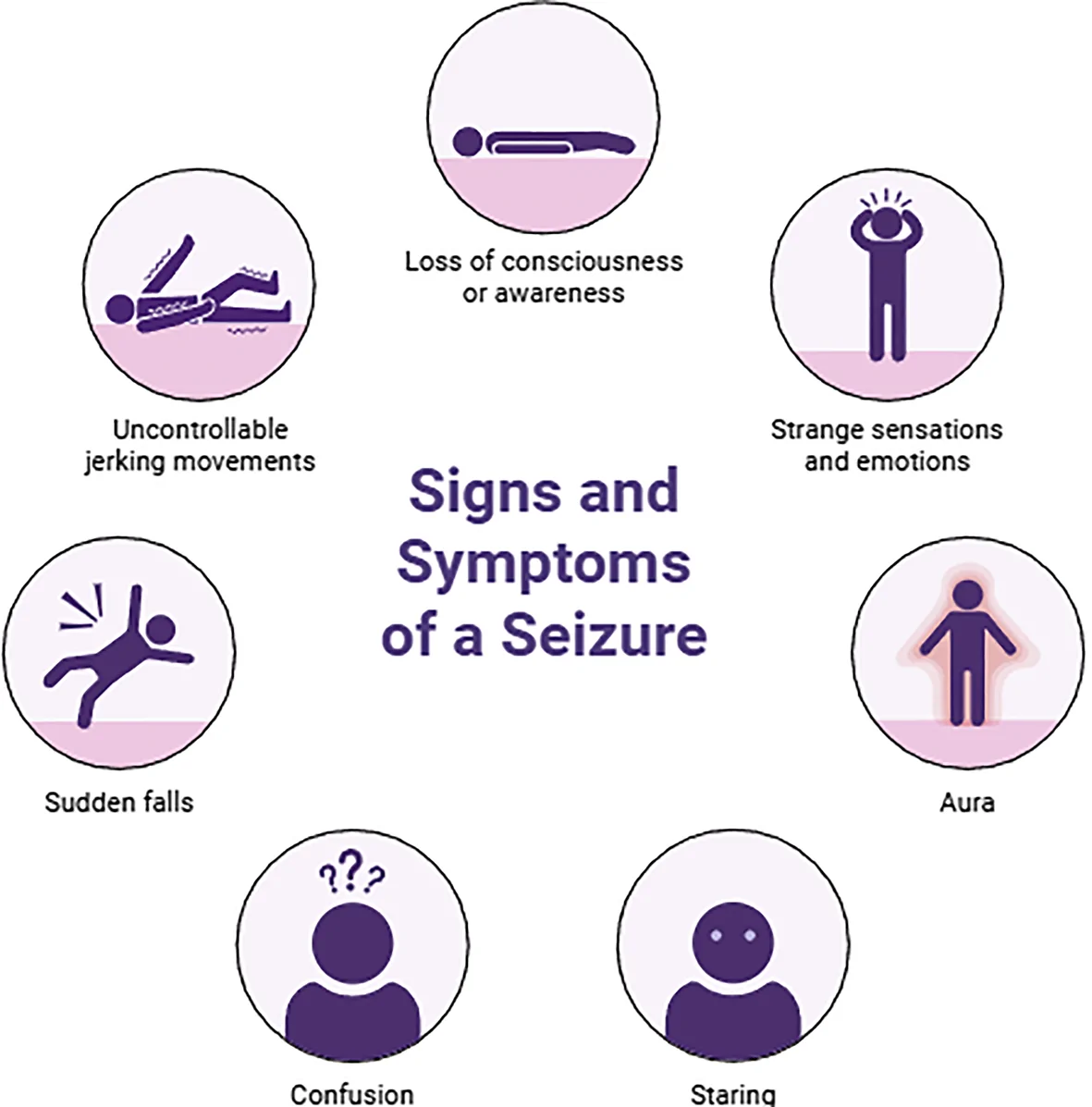
Signs and symptoms of a seizure

**Fig. 3:**
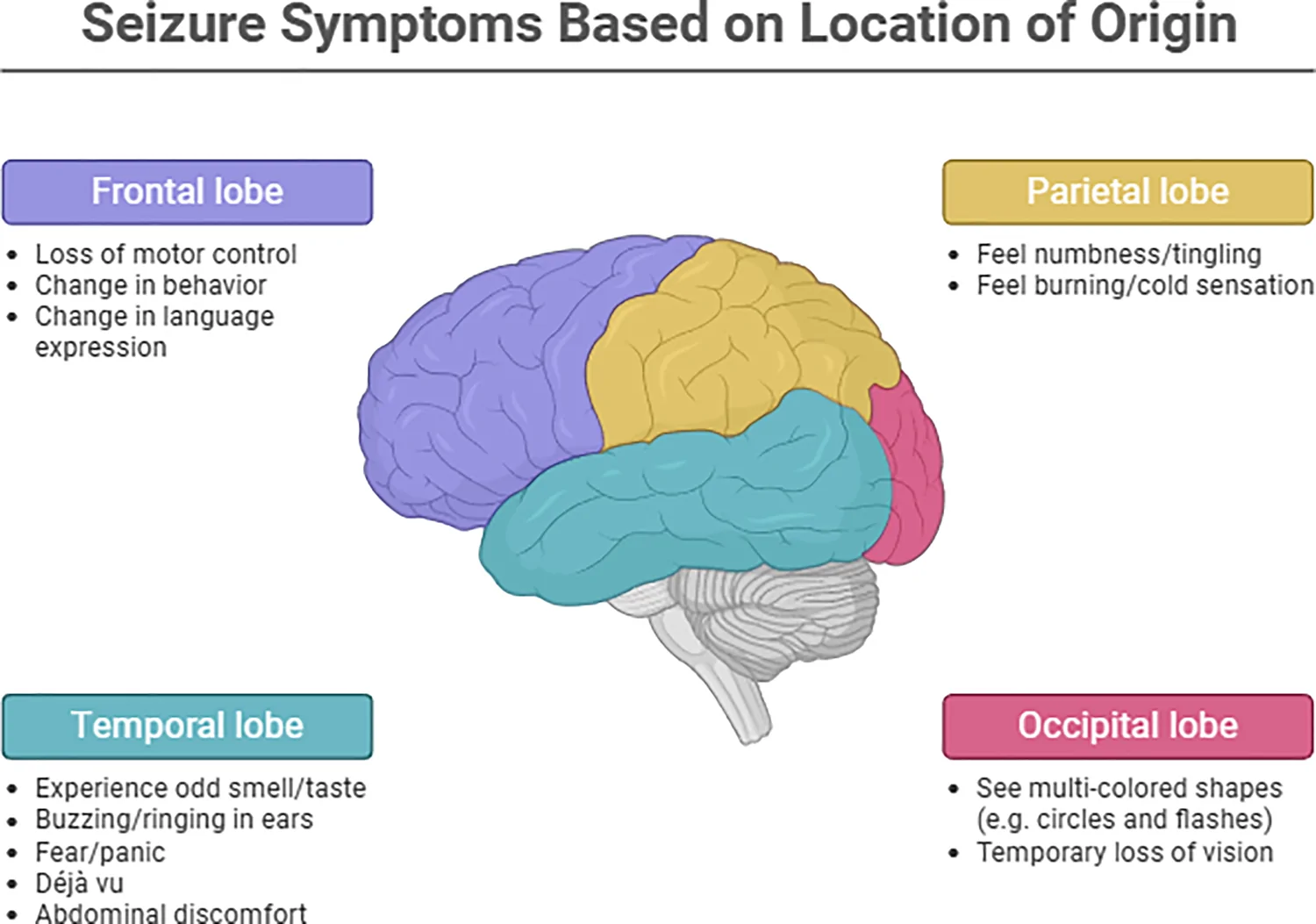
Seizure symptoms based on location of origin

Seizures present a wide range of clinical symptoms, including motor/twitching or convulsions, sensory changes, cognitive dysfunction, and altered awareness. Figures 2 and 3 demonstrate the vast nature of seizure symptoms, showing the array of both motor and non-motor clinical presentations that can make timely recognition and identification difficult. Furthermore Fig. 3 demonstrates the symptoms based on the location of origin. Figure 3 shows that seizures can manifest in many ways depending on the location of their cortical origin and can influence the motor, sensory, cognitive, or behavioral functions of the patient. Associating the symptoms to affected brain regions can provide useful clinical information regarding seizure localization and diagnosis.

**Fig. 4:**
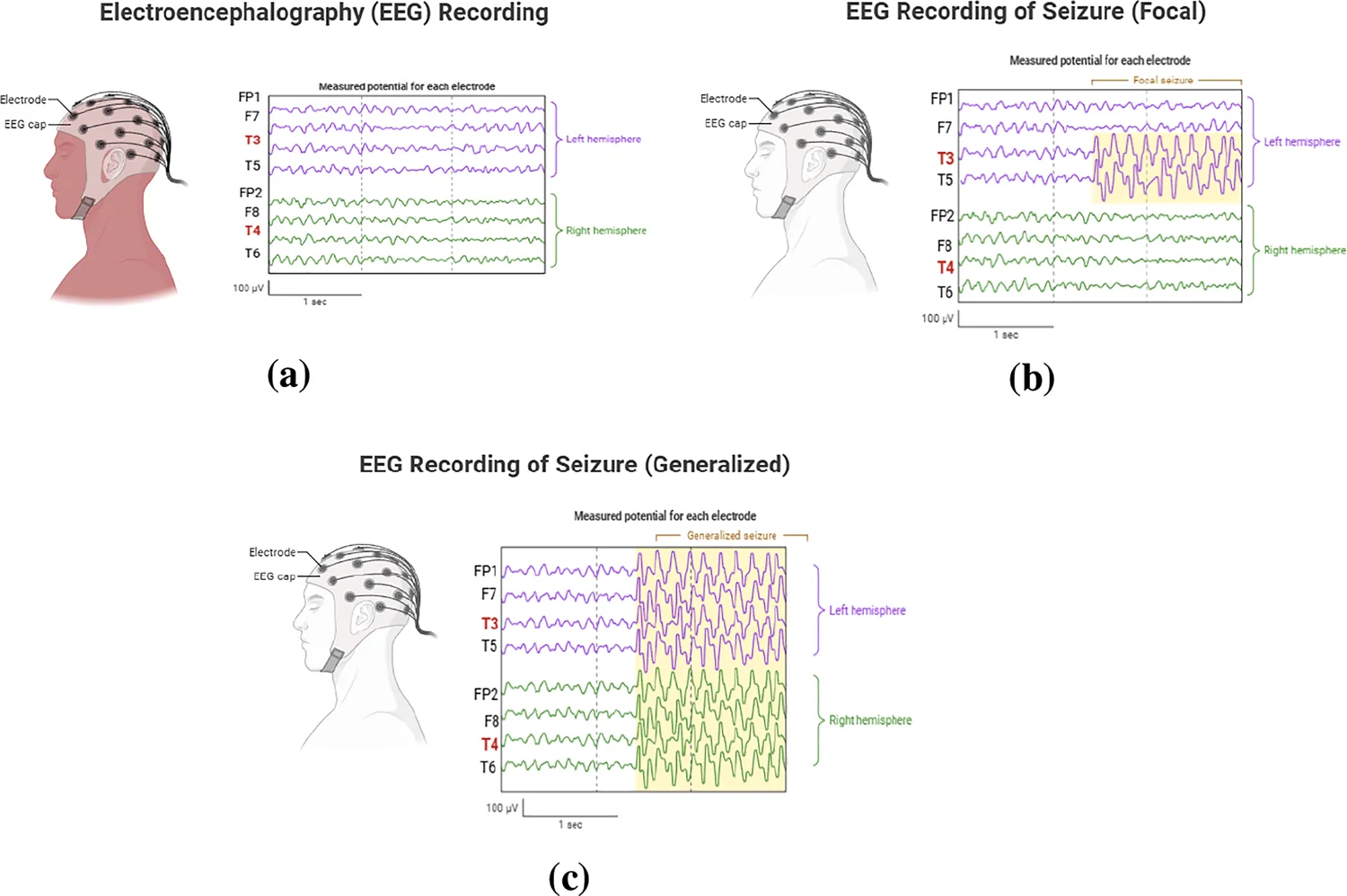
(**a**) EEG recording showing normal brain activity with electrode placement and measured potentials across left and right hemispheres (**b**) recording of a focal seizure event, highlighting abnormal electrical activity localized in the left hemisphere (T3 channel) (**c**) EEG recording of a generalized seizure, illustrating widespread abnormal activity affecting both hemispheres simultaneously

Electroencephalography (EEG) remains the main method for seizure detection and classification because it reveals direct insights into electrical dynamics in the brain. Figure 4a depicts the common EEG acquisition system found within clinical practices, where electrodes placed on the scalp record spatiotemporal brain dynamics. EEG presents a non-invasive, cost-efficient option for seizure monitoring but includes complexities and challenges in interpretation due to factors such as artifacts, interpatient variability, and the non-linear and non-stationary characteristics of the signals. Addressing these challenges may necessitate the use of more advanced and novel signal processing and machine learning approaches, including the proposed VMD-based, multi-domain method developed in this work.

### EEG dataset and characteristics

In this research, we used the TUSZ v2.0.3 dataset, which is one of the most complete and commonly accessed open-access EEG seizure datasets. The repository contains scalp EEG datasets and annotations on seizure events that are long enough to conform with multi-class classification tasks that relate to clinical relevance. The TUSZ dataset,contains various collective demographic groups aged, and both males and females to ensure variability among subjects. This variation is essential for the repeatability and generalizability of automated seizure classification frameworks, as previously noted in works using the TUSZ.

Experienced neurologists have annotated the raw EEG signals from TUSZ, which contain various seizure types [32]. In brief, Table 3 summarizes the seizure types included in the research and their corresponding EEG recordings. This classification serves as an organized starting point for seizure acknowledgment across nine classes while also addressing the reality of variances in seizure manifestations within a clinical context. Unlike single-channel datasets such as Bonn University EEG [8] or datasets focused on pediatrics like CHB-MIT [7], the TUSZ corpus allows multi-class real-world seizure investigation, making it the most logical choice for evaluating the proposed VMD-based multi-domain framework.

**Table 3:**
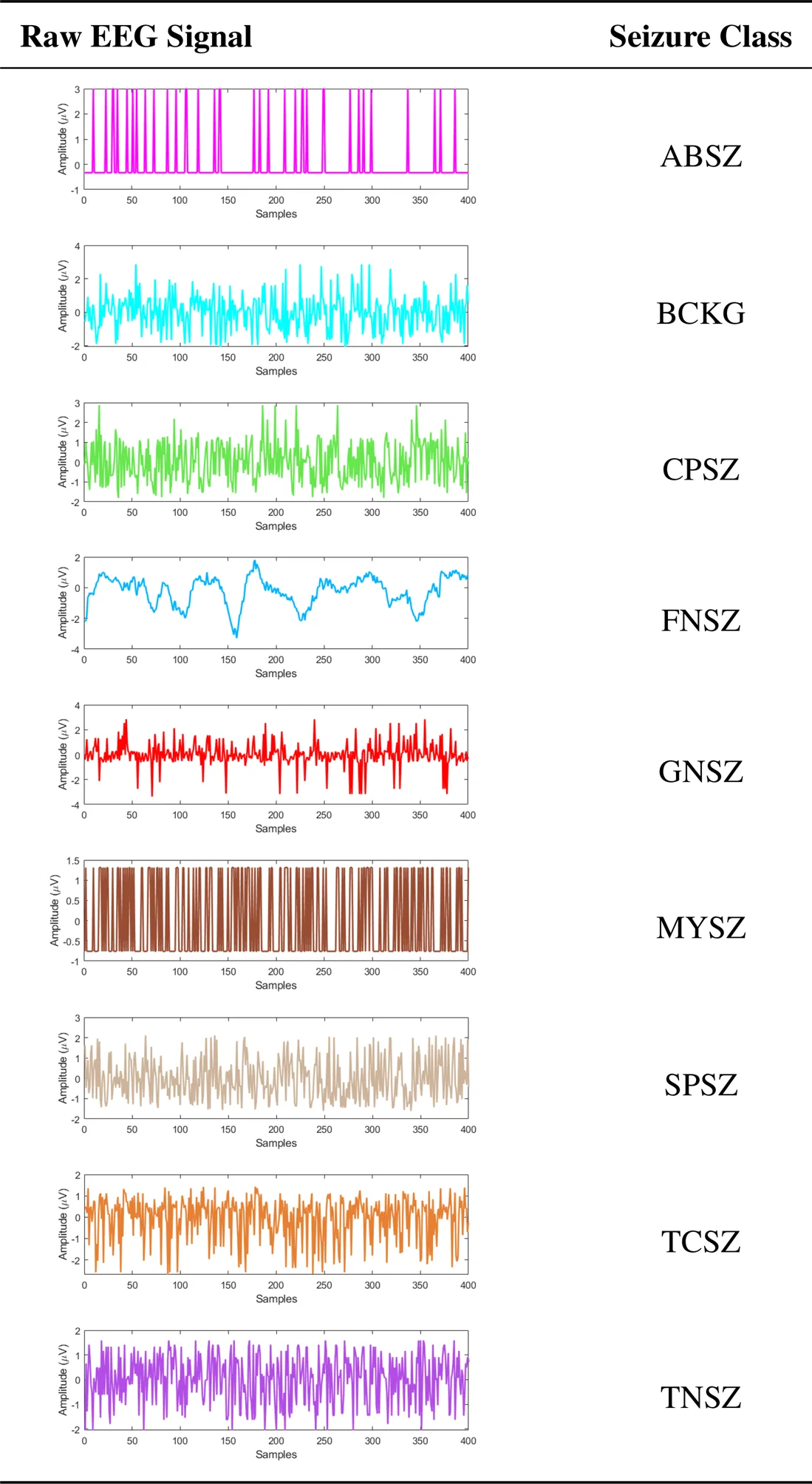
Seizure type annotations and corresponding EEG recordings in TUSZ

**Fig. 5:**
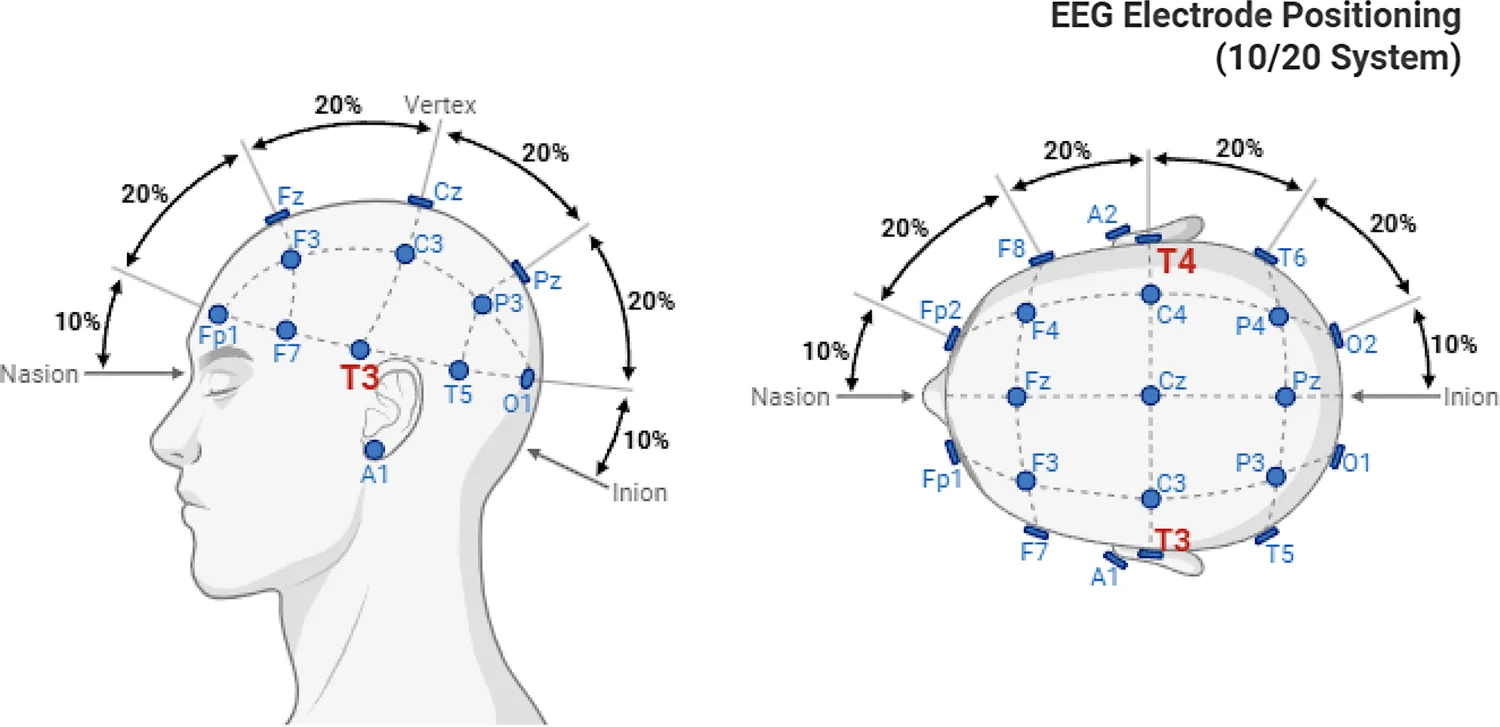
Electrode positioning in the 10–20 EEG system, highlighting the temporal channels T3 and T4

The dataset was registered using the global 10–20 EEG electrode placement system in Fig. 4. For the analysis, we were focused on two channels located at the temporal lobes (T3 and T4) because they are the areas that most commonly originate seizures and provide biologically meaningful information for a classification model. Normal EEG recordings from TUSZ are displayed in Fig. 4a , where the signal from the left hemisphere and right hemisphere exhibited stable rhythmic activity. Figure 4b, followed by TUSZ, illustrated that localized abnormal discharges occurred during one focal seizure event in the left hemisphere and that widespread synchronized abnormal discharges were displayed by both hemispheres during a generalized seizure in Fig. 4c. These examples demonstrated the variety of ways seizures can present and the need for sufficient feature extraction to obtain reliable classification from even well-known events. Also, Fig. 5 shows the electrode placements, highlighting our temporal T3 and T4 channels.

In summary, TUSZ dataset Table 4 has many benefits, such as multi-class seizure bands, demographic variation, and clinical-quality labels. There are, however, substantial challenges such as class imbalance, inter-patient variability, and artifacts, which are noted in some previous work [9, 11]. To address these challenges, we performed preprocessing and synthetic oversampling methods (SMOTE) before feature extraction, allowing for balanced and representative learning from the seizure classes.

**Table 4:** Key highlights of TUSZ dataset for this study

Aspect	Description
Dataset Source	TUSZ v2.0.3
Scope	Multi-class seizure classification with 9 seizure types
Subjects	Diverse demographics (age, gender, medical conditions)
Seizure Types	Clinically annotated by neurologists Table 3
Recording Modality	Scalp EEG using International 10–20 System Fig. 4a
Focus Channels	Temporal channels T3 and T4 (clinically relevant seizure origin sites)
Advantages	Large-scale, real-world clinical recordings, diverse seizure coverage
Challenges	Class imbalance, inter-patient variability, artifacts (addressed with SMOTE)

## Materials and methods

### Proposed hybrid LightGBM–Logistic Regression framework for seizure classification

**Fig. 6:**
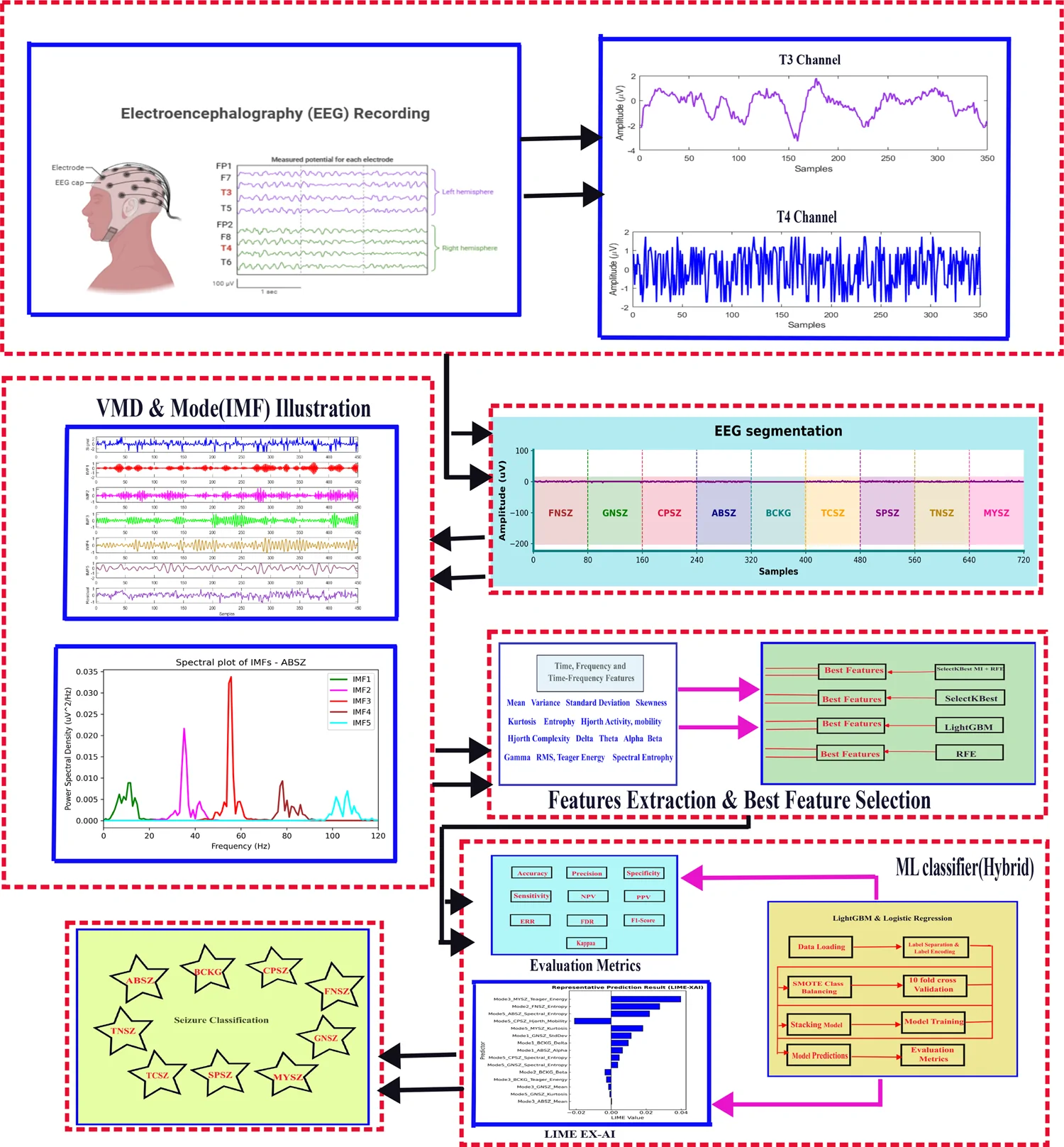
Proposed hybrid LightGBM–Logistic Regression framework with VMD for EEG-based seizure classification

Figure 6 shows the proposed EEG-based seizure classification approach using VMD to decompose EEG signals into informative modes. Since the EEG records from the TUSZ database had not been standardized and we know that not all seizure types will yield the same EEG reading, preprocessing was applied to discard the high-frequency noise and avoid artifacts. EEG segments were extracted using a windowing structure to extract both focal and generalized seizure information. The modes were decomposed,and time, frequency,and time-frequency features were extracted,which became the full feature pool. As a means of reducing the feature space, feature selection techniques were then applied to each feature using SelectKBest MI-RFE, SelectKBest MI, LightGBM importance score, and RFE to extract the most discriminative features. The features were then used to build a hybrid LightGBM-Logistic Regression model, where the former captured complex patterns and the latter improved generalization and interpretation. The potential techniques applied to address the class imbalance observed were SMOTE and validated to avoid overfitting. Lastly, a comparison was performed at each level of task complexity to establish the baseline classifiers, while implementation details are highlighted in the next sections.

### Decomposition of EEG signals into band-limited modes using VMD

VMD is an adaptive signal decomposition technique that is well-suited for the study of nonlinear and non-stationary signals like electroencephalogram (EEG) data. In the present study, VMD is used to decompose the seizure EEG signals into a set of band-limited IMFs [33, 34] that are amplitude-modulated and/or frequency-modulated oscillatory components. According to the proposed system, the number of VMD modes has been determined by trial-and-error methods to be five for effective representation, discrimination, and computation efficiency. According to preliminary investigation, the use of few numbers of VMD modes could not provide enough components for decomposing seizure activities, whereas increasing the number of components above five caused redundancy and increased computation cost.The selected five-mode decomposition provided an effective balance between preserving meaningful seizure-related temporal and spectral information and minimizing mode overlap or redundant feature generation. Furthermore, the five BLIMFs enabled the extraction of complementary multi-domain characteristics from low-frequency, mid-frequency, and high-frequency EEG components associated with different seizure dynamics.

It should be noted that VMD is not considered an empirical mode decomposition (EMD) method,as EMD methods commonly suffer from mode mixing, while VMD utilizes a variational optimization framework to iteratively extract each mode at unique centers of frequency that allow for improved stability in the retrieval of phases and robustness to noise. In contrast to EMD, which extracts modes that may interact with one another, VMD enforces a reconstruction constraint on the IMFs by utilizing a Lagrangian multiplier. By enforcing this constraint, the IMFs produce a unique combination that will reconstruct the original EEG signal and allow for simultaneous denoising/decomposition. This makes VMD extremely effective for isolating rhythms of physiologically meaningful brain activity, including delta, theta, alpha, beta, and gamma bands that contribute to the characterization of seizure-related brain activity. In addition to isolating physiologically meaningful oscillatory activity, VMD is computationally efficient, suited for potentially low-complexity seizure monitoring applications, and eases iterative mode updating via frequency domain form.

VMD solves a constrained variational problem, in which the EEG signal can be decomposed into a set of band-limited IMFs and noise.Effectively, this signifies that noise can be separated during decomposition. Therefore, VMD solves the mode mixing issue, which is a concern for EMD and similar decomposition approaches. Subsequently, for an input EEG signal x(t), the constrained optimization we can express our constrained optimization as a minimization problem Eq. 1 with a reconstruction constraint, which is expressed in Eq. 2.

1 $$\min_{\{m_k\},\{\omega_k\}}\left\{\sum_{k=1}^{K} \left\| \partial_t \left[ \left( \delta(t) + \frac{j}{\pi t} \right) * m_k(t) \right] e^{-j \omega_k t} \right\|_{2}^{2} \right\}$$

2 $$ \sum_{k=1}^{K} m_k(t) = x(t)$$

Eq. (1) represents the constrained variational optimization problem used in Variational Mode Decomposition (VMD), while Eq. (2) defines the signal reconstruction constraint. In these equations, *x(t)* denotes the original EEG signal, $m_k(t)$ represents the $k^{th}$ decomposed mode function, $\omega_k$ indicates the center frequency associated with the $k^{th}$ mode, and *K* denotes the total number of decomposed modes. The symbol *δ(t)* represents the Dirac delta function, *j* denotes the imaginary unit, * indicates the convolution operation, and $\partial_t$ represents the partial derivative with respect to time. The term $\|\cdot\|_2^2$ denotes the squared $L_2$ norm used to estimate the bandwidth of each decomposed mode.

In tackling this constrained problem, VMD converts the problem into an augmented Lagrangian form Eq. 3. A quadratic penalty factor *β* and a Lagrangian multiplier *λ*. The solution was subsequently computed by using the Alternate Direction Method of Multipliers (ADMM) algorithm to update the IMFs and their centre frequencies.

3 $$\begin{aligned}L(\{m_k\}, \{\omega_k\}, \lambda)&= \beta \sum_{k=1}^{K}\left\|\partial_t\left[\left(\delta(t) + \frac{j}{\pi t}\right)* m_k(t)\right]e^{-j\omega_k t}\right\|_2^2 \\& +\left\|x(t) - \sum_{k=1}^{K} m_k(t)\right\|_2^2 \\& +\left\langle\lambda(t),x(t) - \sum_{k=1}^{K} m_k(t)\right\rangle\end{aligned}$$

Eq. (3) represents the augmented Lagrangian formulation used in the VMD optimization process. In this equation, $L(\{m_k\}, \{\omega_k\}, \lambda)$ denotes the augmented Lagrangian function, *β* represents the quadratic penalty parameter controlling reconstruction fidelity, and *λ(t)* denotes the Lagrange multiplier used to enforce the reconstruction constraint. The term $\left\langle \cdot,\cdot \right\rangle$ represents the inner product operation. All other symbols retain their previously defined meanings from Eq. (1) and Eq. (2).

Specifically, In particular, the update step for the IMF is found in Eq. 4, and the center frequency for each mode is again recalculated during each iteration as seen in Eq. 5.

4 $$\hat{m}_{k}^{n+1}(\omega) = \frac{\hat{x}(\omega) - \sum_{i \neq k} \hat{m}_{i}(\omega) + \frac{\hat{\lambda}(\omega)}{2}} {1 + 2 \beta (\omega - \omega_k)^2}$$

5 $$\omega_k^{n+1} = \frac{\int_{0}^{\infty} \omega \, |\hat{m}_k(\omega)|^2 \, d\omega} {\int_{0}^{\infty} |\hat{m}_k(\omega)|^2 \, d\omega}$$

Eq. (4) updates the decomposed mode function in the frequency domain during the iterative VMD optimization process, whereas Eq. (5) updates the center frequency associated with the corresponding mode. In these equations, $\hat{m}_{k}^{n+1}(\omega)$ represents the updated frequency-domain representation of the $k^{th}$ mode at iteration *(n+1)*, $\hat{x}(\omega)$ denotes the Fourier transform of the input EEG signal, and $\hat{\lambda}(\omega)$ represents the Fourier-domain Lagrange multiplier. The parameter $\omega_k$ denotes the center frequency of the $k^{th}$ mode, while *ω* represents the angular frequency variable. The notation $|\hat{m}_k(\omega)|^2$ indicates the power spectrum of the decomposed mode in the frequency domain.

To obtain an accurate recontracting, the dual variable is updated with each update step using the dual ascent method as specified in Eq. 6.

6 $$\hat{\lambda}^{\,n+1}(\omega) = \hat{\lambda}^{\,n}(\omega) + \tau \left( \hat{x}(\omega) - \sum_{k=1}^{K} \hat{m}_{k}^{\,n+1}(\omega) \right)$$

Eq. (6) represents the iterative update of the Lagrange multiplier used to enforce the signal reconstruction constraint during the VMD optimization process. In this equation, $\hat{\lambda}^{\,n+1}(\omega)$ and $\hat{\lambda}^{\,n}(\omega)$ denote the updated and previous Fourier-domain Lagrange multipliers, respectively, while *τ* represents the update step size parameter. The term $\hat{x}(\omega)$ denotes the Fourier transform of the original EEG signal, and $\hat{m}_{k}^{\,n+1}(\omega)$ represents the updated frequency-domain decomposed mode obtained at iteration *(n+1)*.

The iterations continue until convergence is reached and our criteria for convergence is defined by the stopping criteria in Eq. 7 as injection the difference in the IMF estimates is below a stoping threshold.

7 $$\sum_{k=1}^{K} \left\| \hat{m}_{k}^{\,n+1} - \hat{m}_{k}^{\,n} \right\|_{2}^{2} < \epsilon$$

Eq. (7) represents the convergence criterion used in the iterative VMD optimization process. In this equation, $\hat{m}_{k}^{\,n+1}$ and $\hat{m}_{k}^{\,n}$ denote the updated and previous frequency-domain decomposed modes of the $k^{th}$ component, respectively, while *ε* represents the predefined convergence tolerance threshold. The iterative decomposition process terminates when the difference between successive mode updates becomes sufficiently small.

The output of the VMD process is a set of IMFs and a residual which describes the reconstruction of the original EEG signal. More relevantly, these IMFs are associated with physiological oscillatory bands (delta, theta, alpha, beta, gamma), making VMD a successful method for the analysis of seizure-related EEG data.

**Figure:**
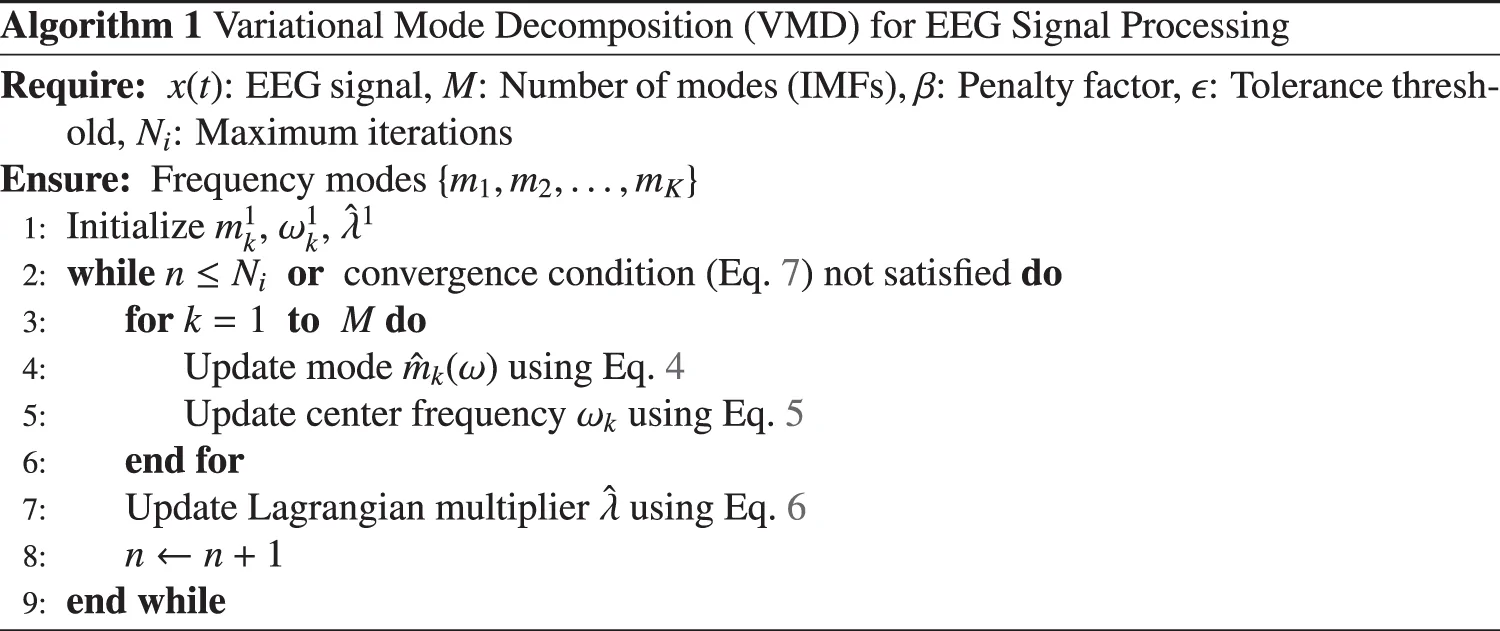

### Feature extraction

In this research, 769 features were extracted from decomposed EEG signals in time, frequency, and time–frequency domains, as shown in Table 5. Mode-wise features such as Mode1_FNSZ_Mean, Mode2_FNSZ_Mean, etc., were calculated from each IMF derived with VMD to maintain multi-resolution signal dynamics. Time-domain features (mean, variance, skewness, kurtosis, entropy, and Hjorth parameters) measure statistical fluctuations and signal complexity, whereas frequency-domain features (delta, theta, alpha, beta, and gamma) reflect spectral content of brain activity closely linked to seizure onset. Moreover, time–frequency features (RMS energy, Teager energy, and spectral entropy) describe transient oscillatory patterns, allowing for a more informative representation of seizure states. Then these features are reduced by selecting the feature with a high mode(IMF)value. Initially, features were extracted from all five VMD-derived modes to preserve complementary temporal, spectral, and nonlinear seizure characteristics. The reference to the mode with the highest frequency was used only for dominant-frequency interpretation and mode-level analysis, not for discarding the remaining modes. Subsequently, the complete multi-mode feature set was processed using mutual information (MI), RFE, and LightGBM-based feature importance ranking to identify the most discriminative features for seizure classification. Thus, the new framework maintains multi-modal data fusion until the feature extraction process, whereas feature selection methods are used later in order to decrease redundancy and select the most informative features from all modalities. Contrary to conventional statistical measures, which are almost insensitive to the non-linearity and non-stationarity properties of EEG recordings, the proposed multi-domain feature space is a source of highly stable and discriminative information for seizure classification [27]. Given the complexity of the resulting high-dimensional feature space, feature selection methods were utilized next in order to select the most informative features.

**Table 5:** Features extracted from EEG signals across different domains

Feature	Domain	Description	Mathematical Notation
Mean	Time Domain	Average amplitude of EEG signal, representing central tendency	$\mu = \frac{1}{N} \sum_{i=1}^{N} x_i$
Variance	Time Domain	Measures the spread of EEG amplitudes around the mean	$\sigma^2 = \frac{1}{N} \sum_{i=1}^{N} (x_i - \mu)^2$
Standard Deviation	Time Domain	Square root of variance; indicates signal dispersion	$\sigma = \sqrt{\frac{1}{N} \sum_{i=1}^{N} (x_i - \mu)^2}$
Skewness	Time Domain	Quantifies asymmetry of the signal distribution	$\mathrm{Skewness} = \frac{\frac{1}{N} \sum_{i=1}^{N} (x_i - \mu)^3}{\sigma^3}$
Kurtosis	Time Domain	Measures peakedness or flatness of the distribution	$\mathrm{Kurtosis} = \frac{\frac{1}{N} \sum_{i=1}^{N} (x_i - \mu)^4}{\sigma^4}$
Entropy	Time Domain	Indicates signal complexity and randomness	$H(X) = - \sum_{i=1}^{N} p(x_i) \log p(x_i)$
Hjorth Activity	Time Domain	Represents the variance of the signal (signal power)	$A = \mathrm{Var}(x(t))$
Hjorth Mobility	Time Domain	Ratio of standard deviation of derivative to signal itself	$M = \sqrt{\frac{\mathrm{Var}(\dot{x}(t))}{\mathrm{Var}(x(t))}}$
Hjorth Complexity	Time Domain	Indicates frequency change; compares mobility to derivative	$C = \frac{M(\dot{x}(t))}{M(x(t))}$
Delta Band Power	Frequency Domain	Power spectral density in *0.5*–*4* Hz range (deep sleep)	$P_\delta = \sum_{f=0.5}^{4} |X(f)|^2$
Theta Band Power	Frequency Domain	Power in *4*–*8* Hz range (drowsiness, early sleep)	$P_\theta = \sum_{f=4}^{8} |X(f)|^2$
Alpha Band Power	Frequency Domain	Power in *8*–*13* Hz range (relaxation states)	$P_\alpha = \sum_{f=8}^{13} |X(f)|^2$
Beta Band Power	Frequency Domain	Power in *13*–*30* Hz range (active thinking)	$P_\beta = \sum_{f=13}^{30} |X(f)|^2$
Gamma Band Power	Frequency Domain	Power above *30* Hz (cognitive/memory functions)	$P_\gamma = \sum_{f > 30} |X(f)|^2$
RMS Energy	Time–Frequency	Root mean square energy; overall signal strength	$E_{RMS} = \sqrt{\frac{1}{N} \sum_{i=1}^{N} x_i^2}$
Teager Energy	Time–Frequency	Instantaneous energy of nonlinear oscillatory signals	$\Psi[x(n)] = x(n)^2 - x(n-1)\,x(n+1)$
Spectral Entropy	Time–Frequency	Quantifies irregularity using normalized spectral power	$H_s = - \sum_{i=1}^{N} P(f_i)\, \log P(f_i)$

### Feature selection

The first suggested method of feature selection in this paper is a hybrid model of SelectKBest Mutual Information with Recursive Feature Elimination (MI-RFE). This model includes using mutual information with SelectKBest as a filter that helps us to keep seizure-related features and then applying recursive feature elimination (RFE) to improve this selected set of attributes. Besides the proposed hybrid feature selection approach, three single feature selection methods were evaluated independently: SelectKBest with Mutual Information, LightGBM Feature Importance, and RFE. This comparison was performed to evaluate the proposed hybrid approach against conventional feature selection methods.

#### SelectKBest MI-RFE

**Fig. 7:**
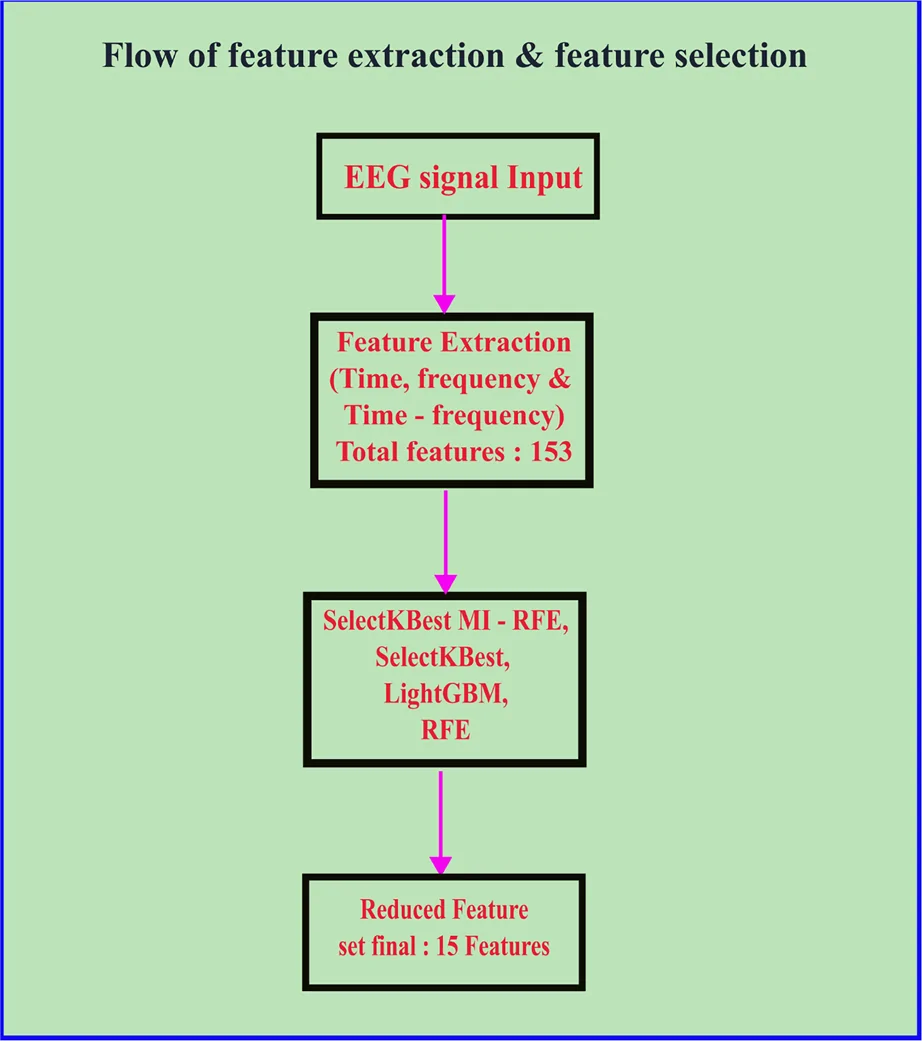
Flow of feature extraction and feature selection: from 153 extracted features to 15 optimal features using domain-specific techniques

This research utilized a two-stage hybrid feature selection approach that incorporated SelectKBest based on Mutual Information (MI) [35] and RFE [36] to further refine the discriminative feature subset created from the initial 153 neural features (Table 5) that were extracted. MI-based SelectKBest finds the features with the most statistical dependence against the seizure type class label. RFE has a wrapper-based iterative process that removes features sequentially based on the results of a selected ML model. In combination, these two approaches, which work well together in choosing the correct features, can be sure that the selected features will be informative. Figure 7 below presents the process of feature extraction and selection that narrows down the number of features from 153 to 15 important features.

Mutual Information is the first-stage filtering method. Given a training set:

8 $$T = \{(x_i, y_i) \mid i = 1, 2, \dots, n \},$$

Where $x_i \in \mathbb{R}^p$ refers to the initial feature vector and $y_i \in \{1, \dots, L\}$ defines the EEG seizure classes, the MI measures the nonlinear dependence between a feature X and the label Y, the MI is calculated as:

9 $$I(X; Y) = \sum_{x \in X} \sum_{y \in Y} p(x, y)\,\log \frac{p(x,y)}{p(x)p(y)},$$

Where p(x,y) indicates the joint distribution, and p(x),p(y) are the marginal distributions. A greater amount of mutual information means the feature is valuable for differentiating between seizure types. By this criterion, SelectKBest ranks all *p* = 153 features and keeps the foremost k candidates:

10 $$\hat{x} = \{x_1, x_2, \dots, x_k \}, \qquad k < p.$$

Nevertheless, MI chooses features independently and doesn’t consider feature redundancy, or interactions between features. To address this limitation the second stage applies RFE using logistic regression as the input estimator. RFE operates by fitting a model in a forward manner and sequentially removing the least important features each time until the optimal number of features has been selected $n_{\mathrm{rfe}}$ is reached. Formally, for a given subset $S \subseteq \hat{x}$ RFE assigns an importance weight vector

11 $$w = (w_{1}, w_{2}, \ldots, w_{k}),$$

and removal of the minimal ranked features in $|w_j|$ until

12 $$|S| = n_{\mathrm{rfe}},$$

To find the best combinations, grid search evaluated multiple MI thresholds $k_{\mathrm{mi}}$ and RFE feature counts $n_{\mathrm{rfe}}$ Each combination was quantified using a 5-fold cross-validation framework with Logistic Regression to identify the pair with the best classification score; this exhaustive search confirmed the final feature set is minimal and maximally discriminative.

**Fig. 8:**
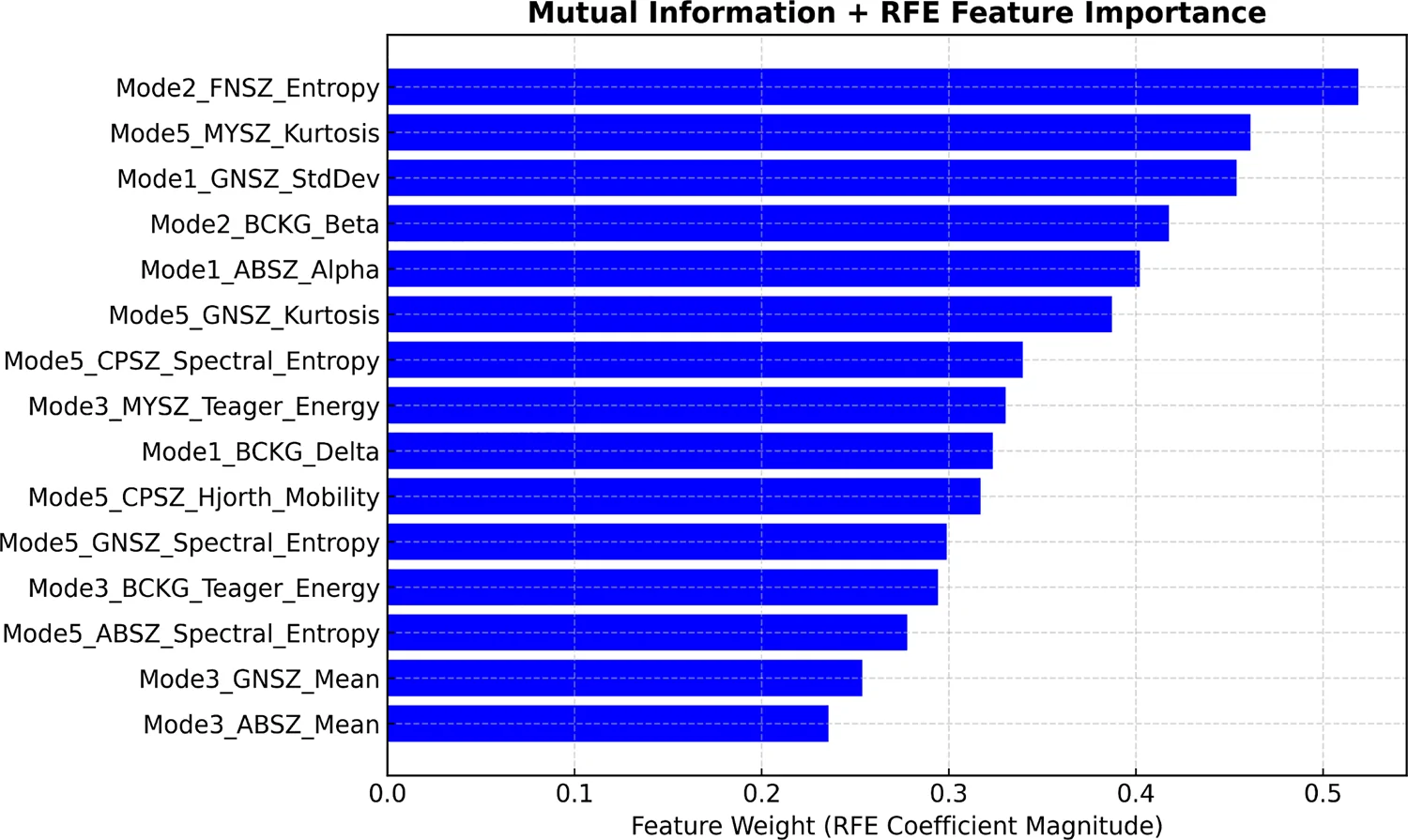
Mutual information + RFE feature importance

In the end, the improved hybrid selection yielded the best performing subset of features, which was then saved as the final reduced feature matrix and was utilized as an input for the proposed Hybrid LightGBM–Logistic Regression architecture. In the Fig. 8, clearly the shows the individual information score.

**Figure:**
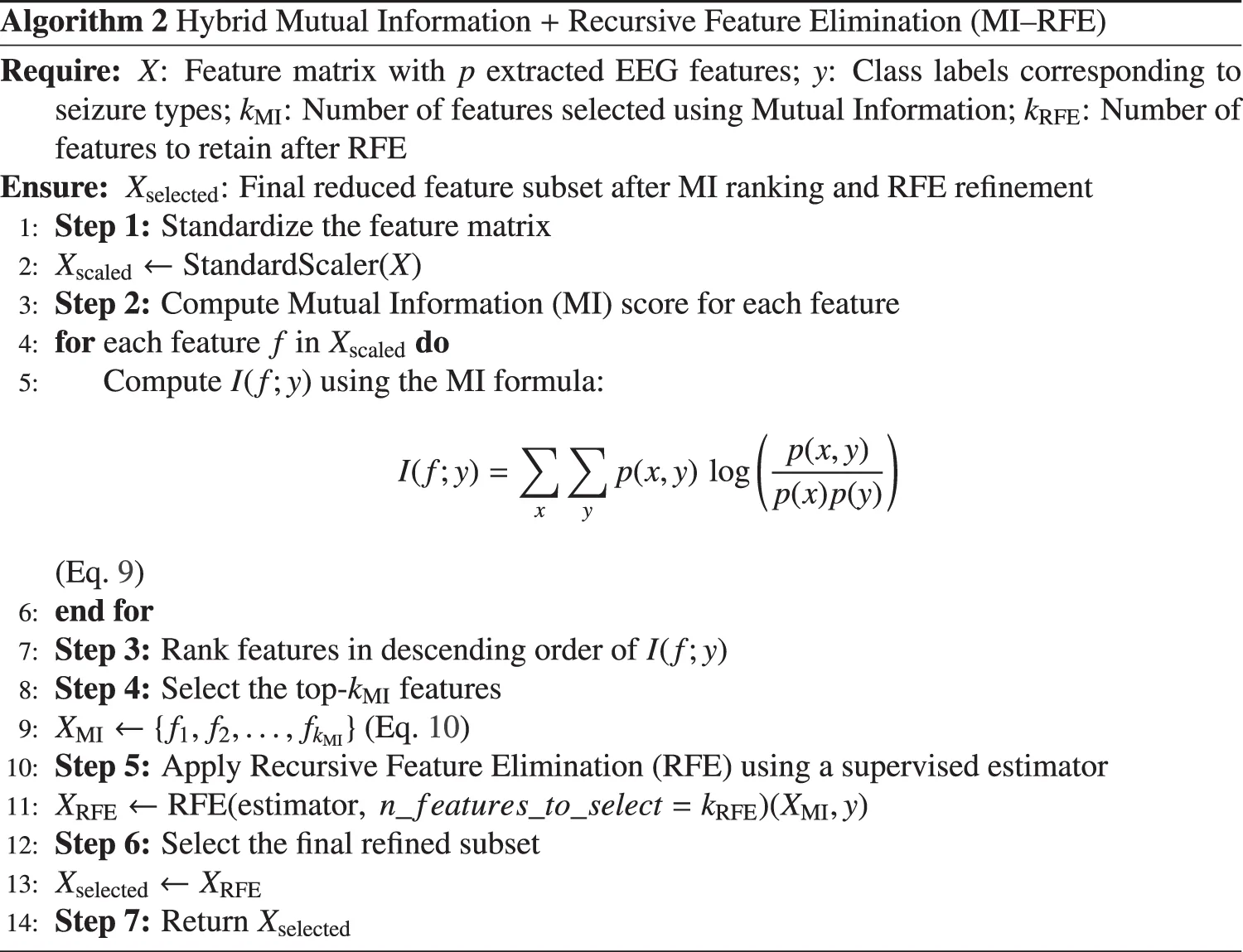

#### SelectKBest with mutual information

In this study, SelectKBest based on Mutual Information was used as a supervised method for feature selection to reduce the dimensionality of the extracted feature space from 89 features Table 5 to the 20 most discriminative features for seizure classification. Mutual Information [37] is a non-parametric criterion that captures statistical dependency between each feature and the target label; hence, it is most suitable for non-linear, non-Gaussian EEG data distributions. Unlike traditional techniques such as PCA (which is unsupervised and ignores class labels) or ReliefF (which is susceptible to noise and redundant features), the MI-based selection checks the relevance of each individual feature concerning seizure types, ensuring that only the most informative attributes are retained.

#### LightGBM-based

LightGBM, a gradient boosting framework [38], ranks features based on their ability to decrease loss when trees are built. It is especially advantageous to EEG seizure classification because it handles a large number of features and can manage imbalanced data sets. It identifies features that improve model accuracy, even in complicated high-dimensional spaces. It is faster than other boosting methods and also less memory-consuming. Therefore, LightGBM importance gives a stable measure of the most contributing features to choose from in EEG data.

#### Recursive feature elimination(RFE)

RFE uses an iterative training model that focuses on the most relevant features in each round, removing the least important variables until the optimal number of features is achieved [39]. By concentrating on ranking features and cutting irrelevant variables in each round, RFE facilitates the removal of all unnecessary variables. RFE [40] adequately cleans and “refines” the feature space and connects to classifier performance easily. This iterative removal of features reduces the chance of overfitting and improves generalization. For EEG data, this approach will help refine relevant features for seizure classification based on subtle differences in brain signals.

#### Class imbalance handling—SMOTE

The TUSZ dataset suffers from a high degree of class imbalance in which some seizure classes have much less data than others. In order to solve this problem without causing any leakage in the data, the SMOTE [41] was used for resampling solely in the training folds in each iteration of cross-validation. Specifically, after stratifying the data using stratified K-fold cross-validation, only the training fold was resampled using SMOTE, whereas the test fold was left untouched. In doing so, no data leakage could happen, since synthetic samples would not be created from the test set, and therefore no information about it would leak into the training phase. Consequently, this approach allows us to improve classifier sensitivity towards rare classes while retaining a robust evaluation procedure at the same time.

#### Two-stage classifier: LightGBM with Logistic Regression

Due to their capacity to manage nonlinear feature interactions, resistance to noise, ability to work with high-dimensional data, and general predictive capabilities in biomedical signal classification tasks, gradient boosting algorithms have emerged as an effective approach. In this paper, we implement a two-stage classification strategy utilizing Light Gradient Boosting Machine (LightGBM) and logistic regression (LR) for multi-class seizure classification. LightGBM is a tree-based, leaf-wise gradient boosting framework that outputs probabilistic class distributions capturing complex relations between EEG features [42, 43]. Logistic regression then uses these probabilities as meta-features to reshape the decision boundary to enhance generalization across patients [44]. The Logistic Regression meta-learner utilized L2 regularization with the default ‘lbfgs’ solver to improve generalization and stabilize learning from correlated probability outputs generated by the LightGBM base classifier.

The LightGBM model minimizes the following objective function:

13 $$L = \sum_i l(y_i, \hat{y}_i) + \sum_k \Omega(f_k)$$

where l(yi, $\hat{\textrm{y}}$i) is the loss function (cross-entropy in this case), and $\Omega(f_k)$ is the regularization term that penalizes the complexity of each weak learner fk. The predicted probability for class c is given as:

14 $$P(y = c \mid x) = \mathrm{softmax}\!\left( \sum_k f_k(x) \right)$$

**Fig. 9:**
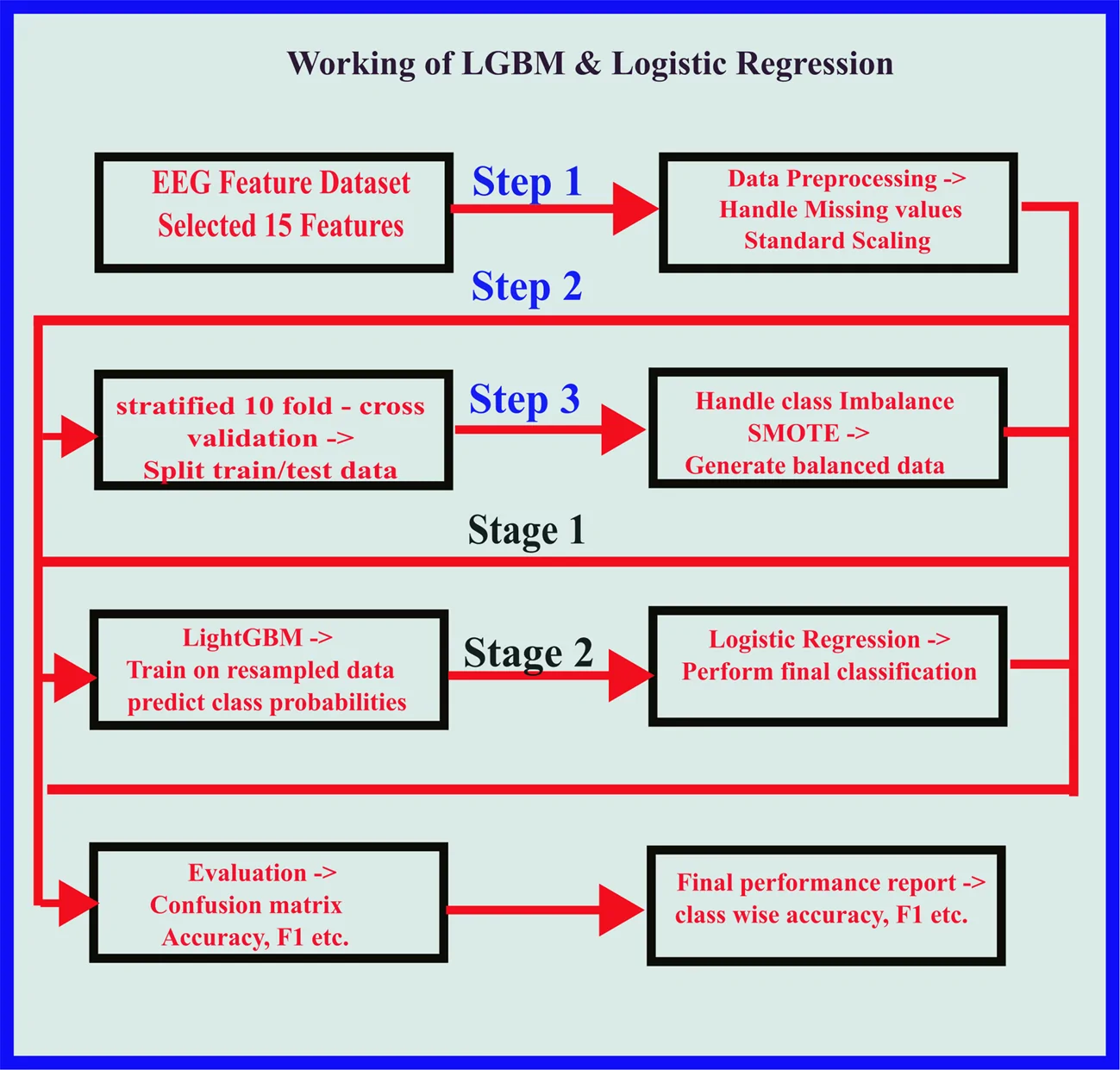
Working of LGBM and Logistic Regression

The resulting probability vectors from LightGBM, $\Pr(y \mid x)$, are then used as input features for the Logistic Regression classifier. Logistic Regression computes the posterior probability of each class as:

15 $$P(y = c \mid P(y \mid x)) = \frac{1}{1 + \exp \!\big( - (w^T P(y \mid x) + b) \big)}$$

where w and b are the weights and bias parameters optimized to maximize the likelihood of correctly classifying seizure types. The principal motivation behind adopting this hybrid framework is the potential to model non-linear EEG dynamics using LightGBM while still being able to leverage the straightforward interpretation of logistic regression for the final prediction. With the two-stage approach, we can minimize overfitting while increasing the stability of predictions based on class imbalance and inter-patient variability. Figure 9 illustrates this process of classification on an EEG dataset by LightGBM and logistic regression.

**Figure:**
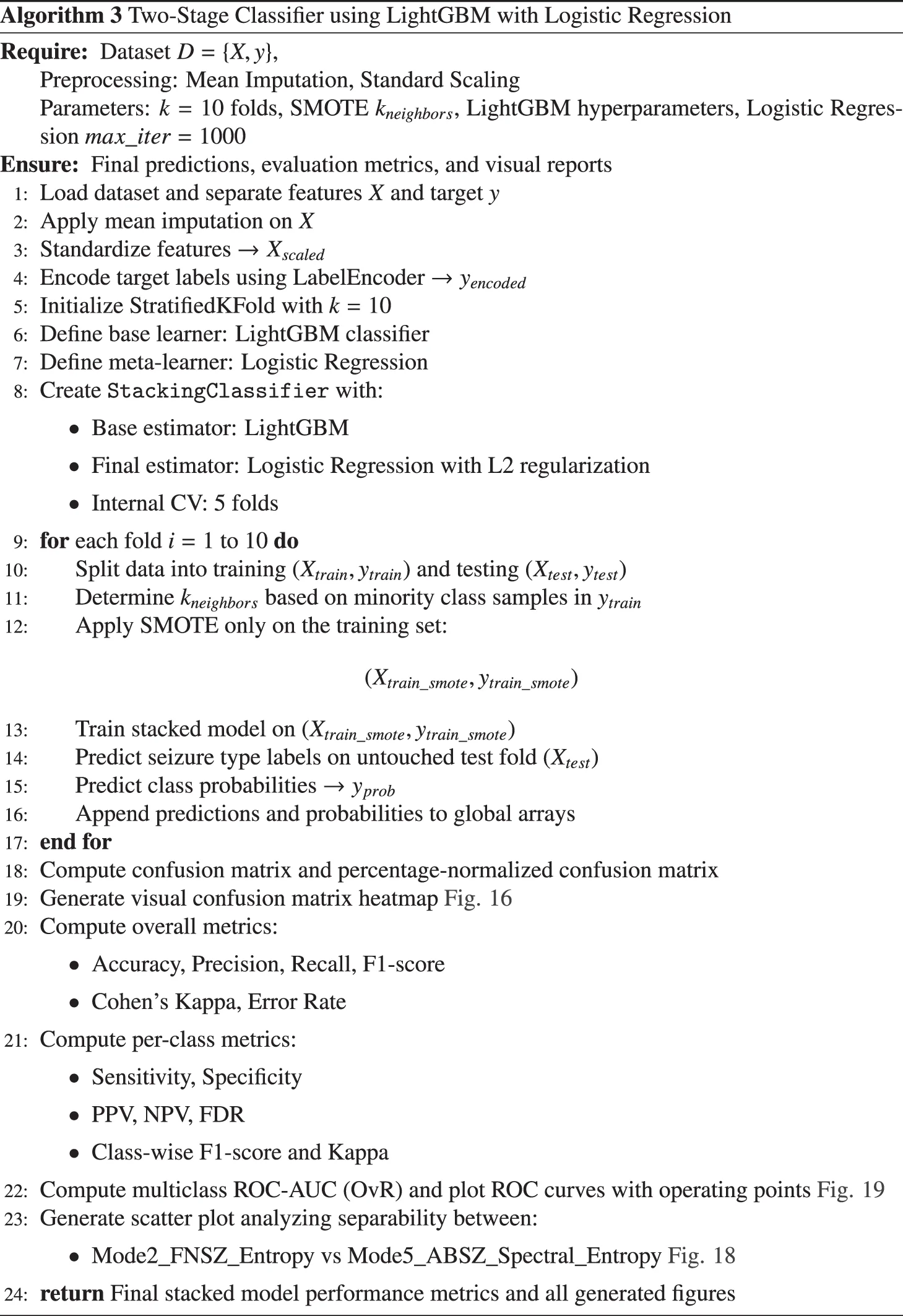

## Results and discussions

**Fig. 10:**
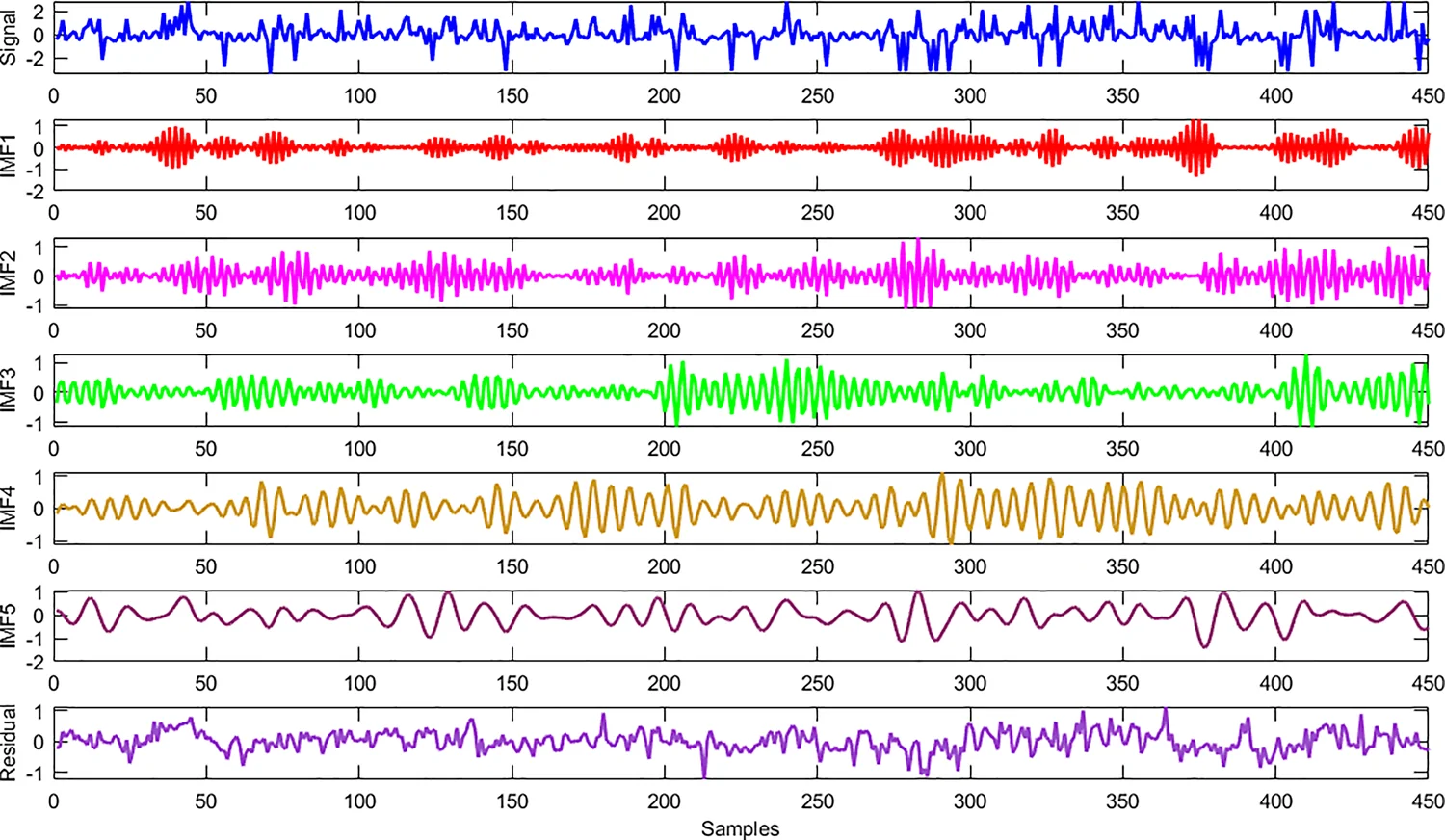
Illustration of EEG signal decomposition into IMFs using VMD

**Fig. 11:**
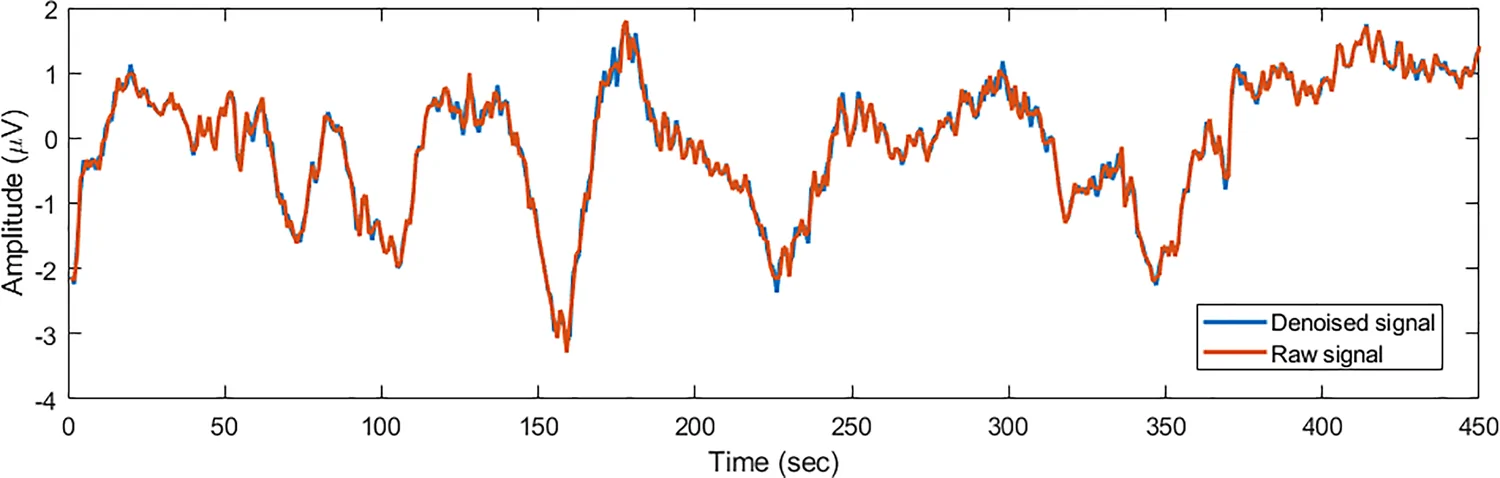
Noise reduction in EEG signals achieved through VMD-based decomposition

**Fig. 12:**
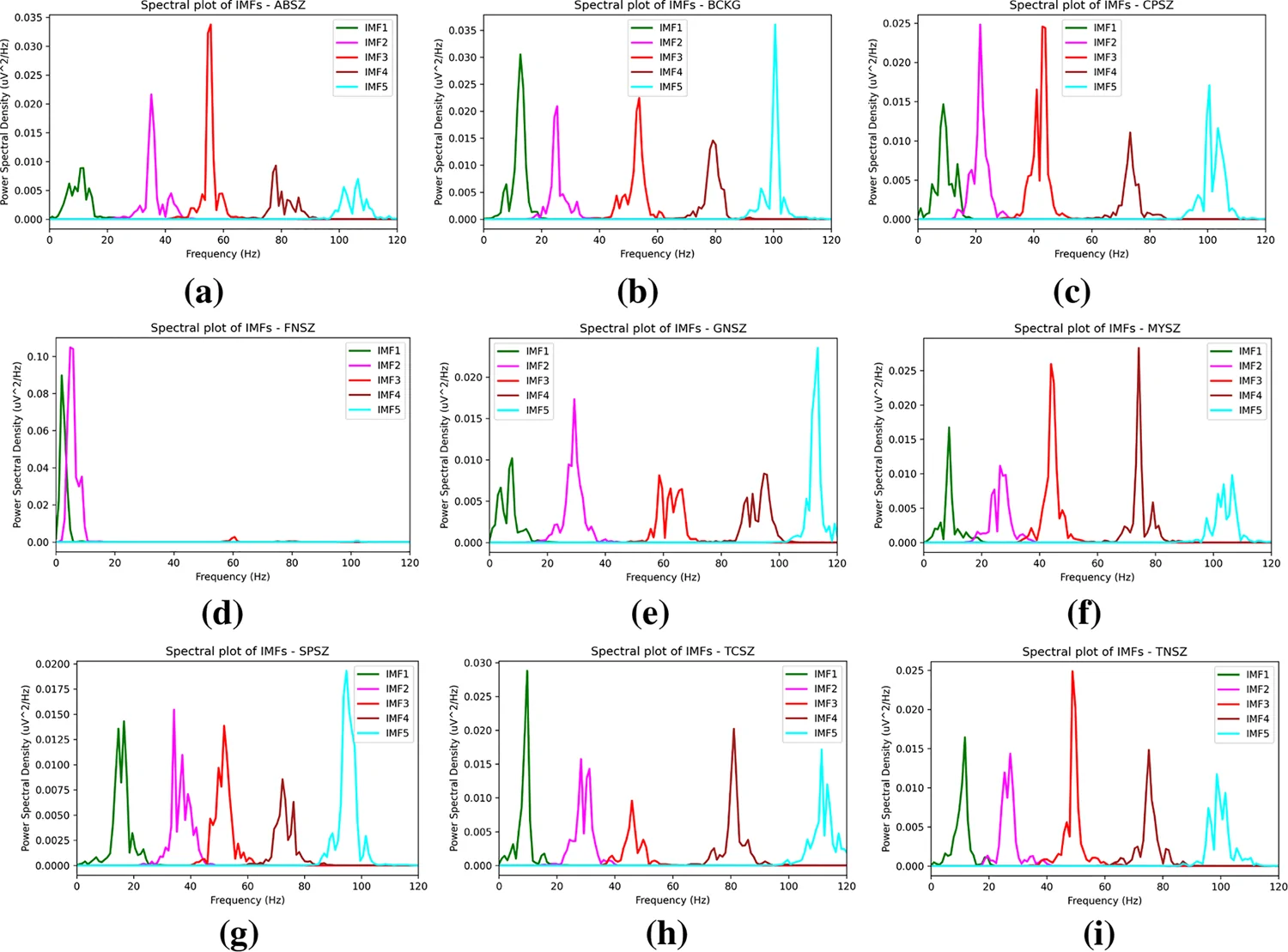
Spectral representation of IMFs obtained from seizure EEG signals (**a**) ABSZ (**b**) BCKG (**c**) CPSZ (**d**) FNSZ (**e**) GNSZ (**f**) MYSZ (**g**) SPSZ (**h**) TCSZ (**i**) TNSZ

The suggested VMD-augmented, two-stage classifier (LightGBM *→* Logistic Regression) for nine-class seizure identification was deployed in Python with scikit-learn and LightGBM on the TUSZ v2.0.3 corpus (24 patients; channels T3/T4). Examination of raw recordings validated the absence of protocol standardization and marked class imbalance between seizure types. Therefore, we initially executed simple preprocessing and windowing-based segmentation to demarcate segments with ictal or inter-ictal activity. VMD was subsequently used as a denoiser and structured decomposer: every EEG segment was decomposed into five intrinsic modes, providing band-limited components that retain clinically relevant rhythms Figure 10 . Suppressing motion contamination and high-frequency artifacts during decomposition, VMD yielded cleaner reconstructions amenable to downstream analysis Fig. 11. Later spectral analysis of the modes identified class-specific power concentrations that drove the choice of information-rich components Fig. 12. Statistical, entropy-based, and spectral features were calculated on all five modes (Modes 1–5) to ensure that both low- and high-frequency dynamics were retained.Now we are taking only the high-frequency IMF range between 5 IMFs in order to focus on the IMF range where frequency spiked high. Then, to eliminate redundancy and keep the most discriminative information, we used a feature selection step (SelectKBest with mutual information – REF), which pruned less informative features and kept those essential for seizure classification. Due to the severe inter-class imbalance in the TUSZ dataset, SMOTE was incorporated within each training fold during stratified K-fold cross-validation. The oversampling process was restricted exclusively to the training partitions, while the test folds remained untouched to avoid data leakage and ensure unbiased performance evaluation.

**Fig. 13:**
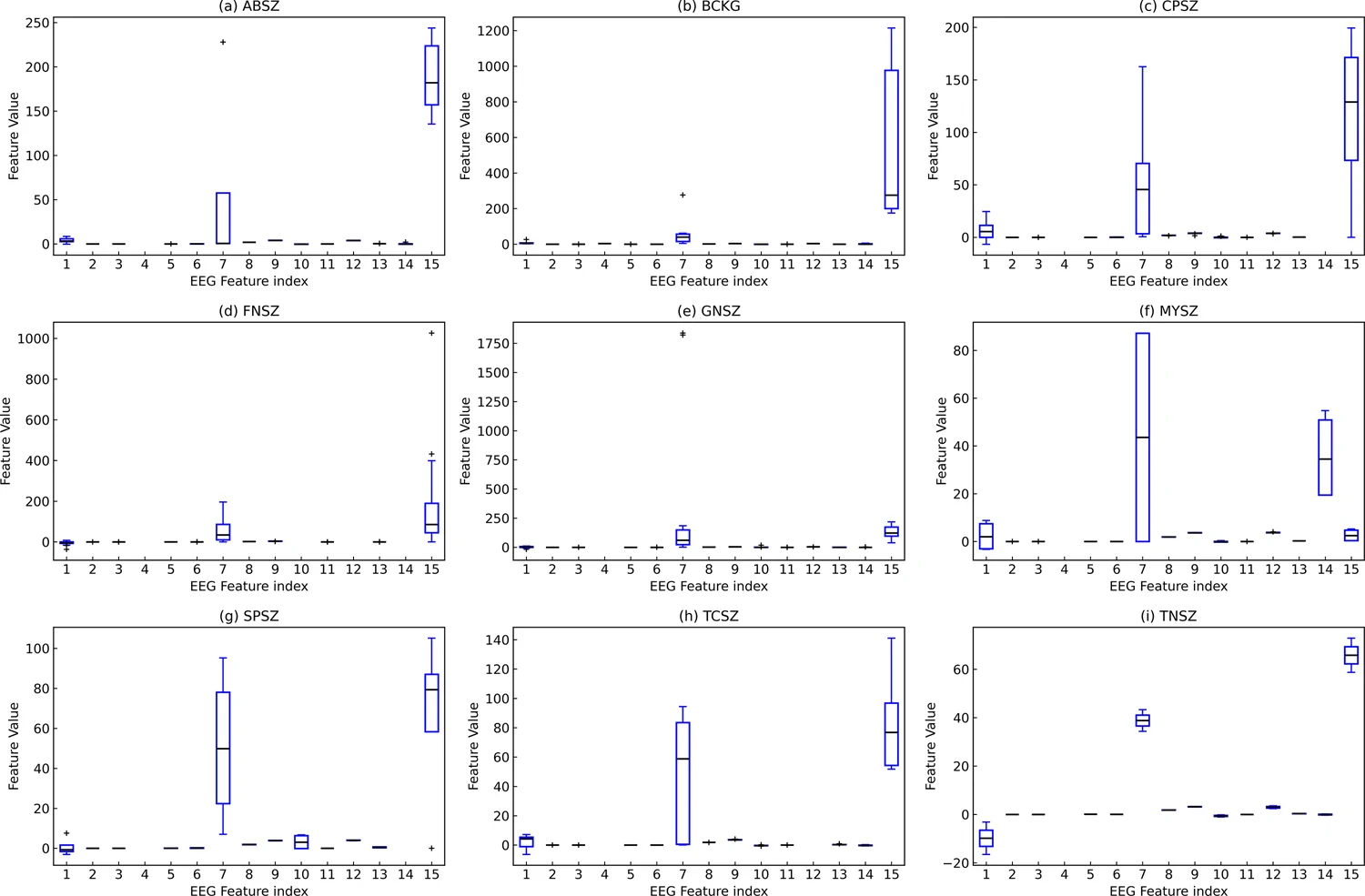
Distribution profiles of features across different seizure classes spectral representation of IMFs obtained from seizure EEG signals (**a**) ABSZ (**b**) BCKG (**c**) CPSZ (**d**) FNSZ (**e**) GNSZ (**f**) MYSZ (**g**) SPSZ (**h**) TCSZ (**i**) TNSZ

**Fig. 14:**
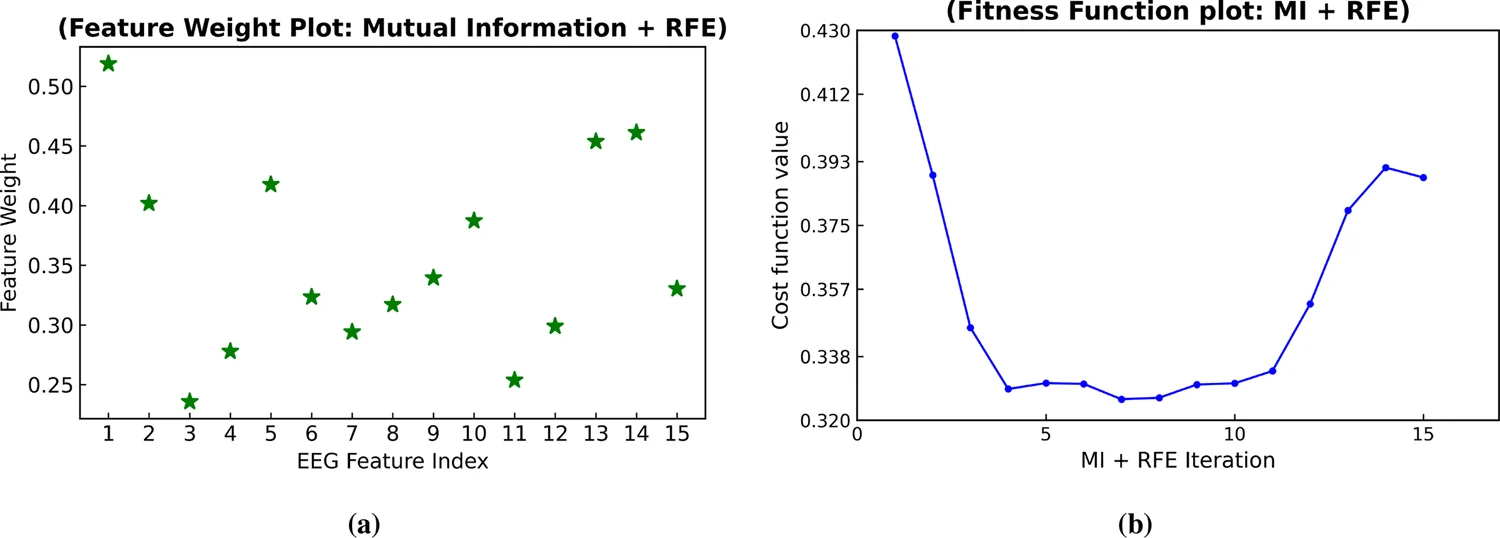
(**a**) Feature weight plot: mutual information + RFE (**b**) fitness function plot: MI + RFE

**Fig. 15:**
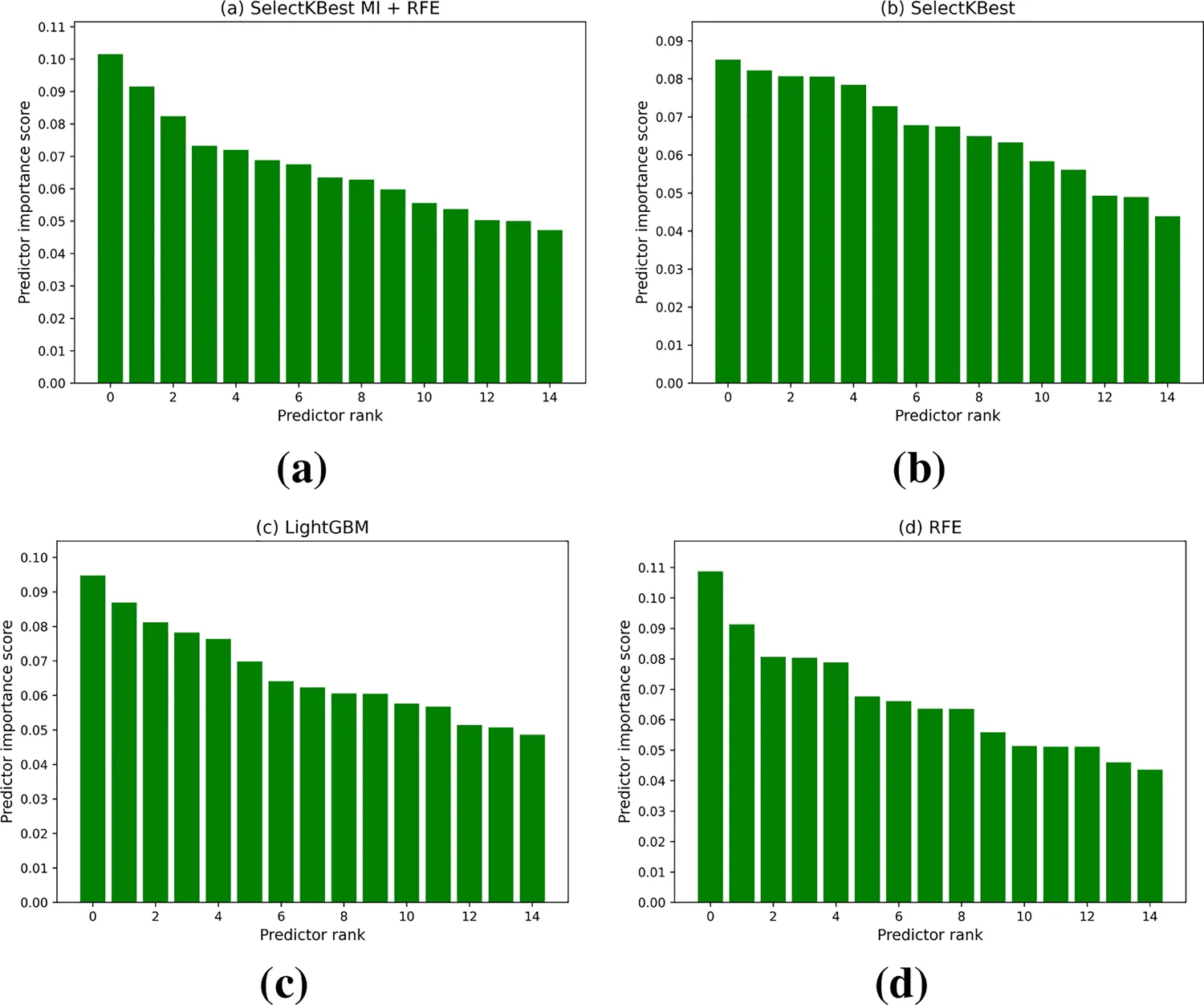
Comparison of predictor importance scores obtained using (**a**) SelectKBest MI-RFE (**b**) SelectKBest (**c**) LightGBM (**d**) RFE

From the five VMD-based modes, a rich set multi domain features Table 5 was derived, and 153 descriptors per EEG segment were obtained. These features represent a wide range of brain dynamics from statistical properties (mean, variance, skewness, and kurtosis) and Hjorth parameters to spectral band powers $(\delta, \theta, \alpha, \beta, \gamma)$ and hybrid measures like RMS energy, Teager energy, and spectral entropy. Figure 13 depicts the variability of representative features, where different patterns across seizure types highlight their discriminative power. For the selection of the most relevant features and elimination of redundant ones, this work utilized four supervised feature selection methods: SelectKBest MI-RFE, SelectKBest MI, LightGBM importance score, and Recursive Feature Elimination (RFE). The impact of individual attributes was initially evaluated by means of SelectKBest MI-RFE, which ranked attributes according to mutual information to eliminate noise-ridden ones. Next, the features are estimated using only mutual information. Next, the tree-based algorithm LightGBM estimated feature contribution in the form of predictor scores, emphasizing consistent features among seizure classes. Then, RFE progressively narrowed down the attribute set by recursively removing the least contributing ones. Comparative examination of feature ranking by these methods Fig. 14a : Feature weights indicating minor features close to zero; Fig. 14b: Fitness curve indicating optimization against key features; Fig. 15: Predictor importance score comparison among selection algorithms) disclosed a strong thresholding strategy. By integrating these strategies, the initial 153 features identified in all five modes were minimized to 15 highly discriminative features with better classification performance and computational efficiency.

### Training and evaluation of classifier models for seizure prediction

**Fig. 16:**
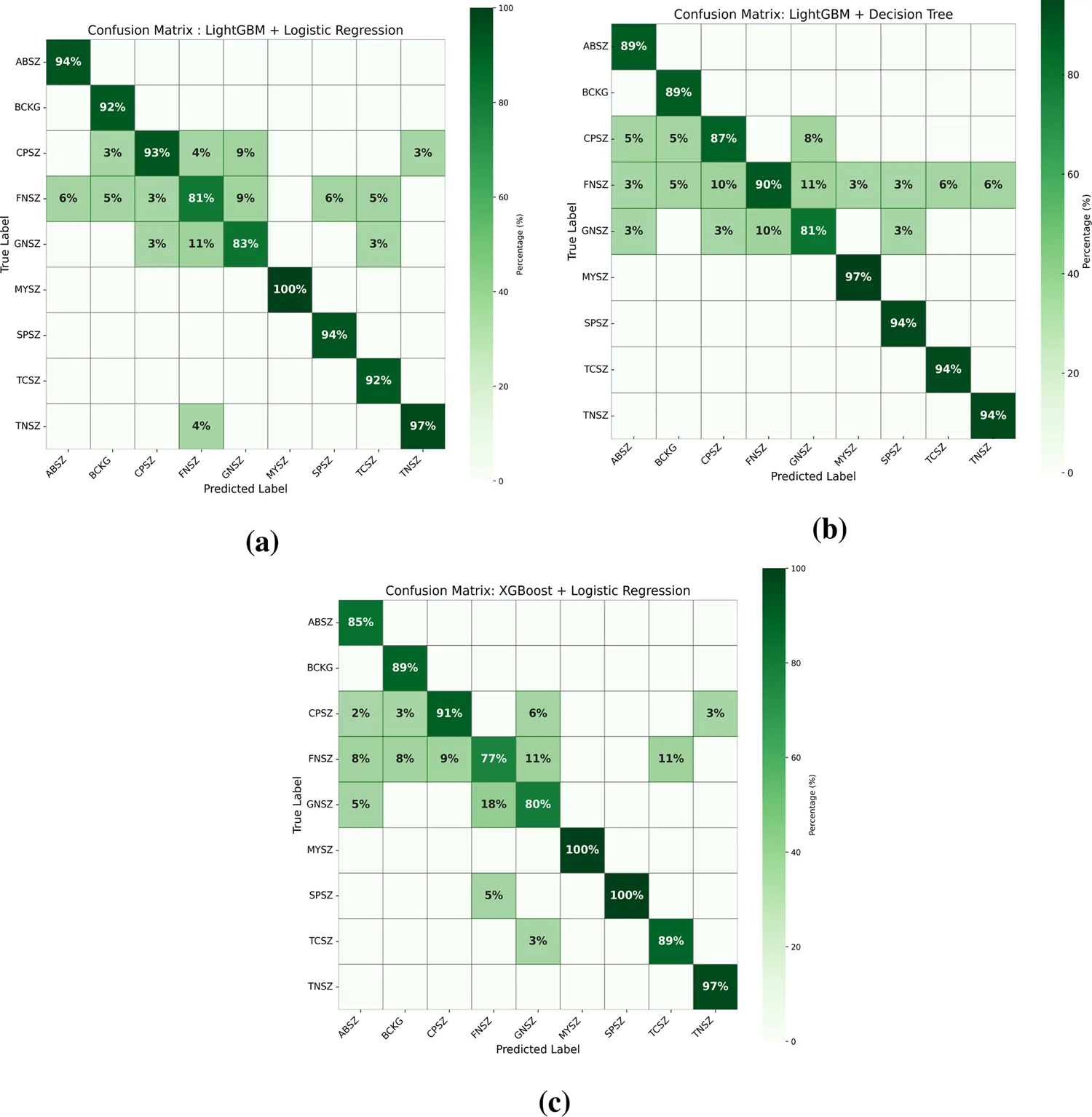
Confusion matrix plots of classifier models (**a**) LightGBM + Logistic Regression (**b**) LightGBM + decision tree (**c**) XGBoost + Logistic Regression

**Fig. 17:**
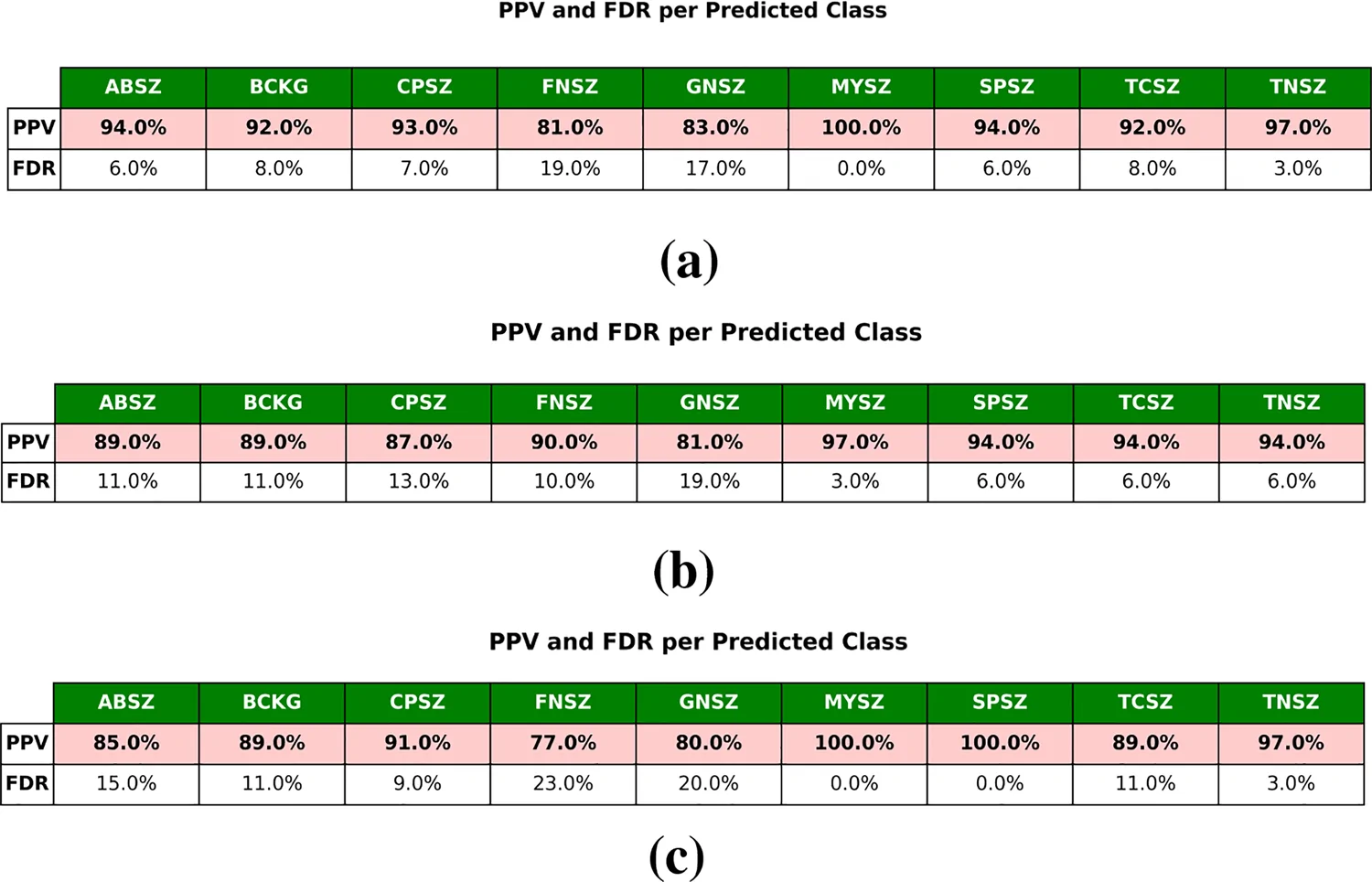
Comparative visualization of positive predictive value (PPV) and false Discovery Rate (FDR) s (**a**) LightGBM + Logistic Regression (**b**) LightGBM + decision tree (**c**) XGBoost + Logistic Regression

**Fig. 18:**
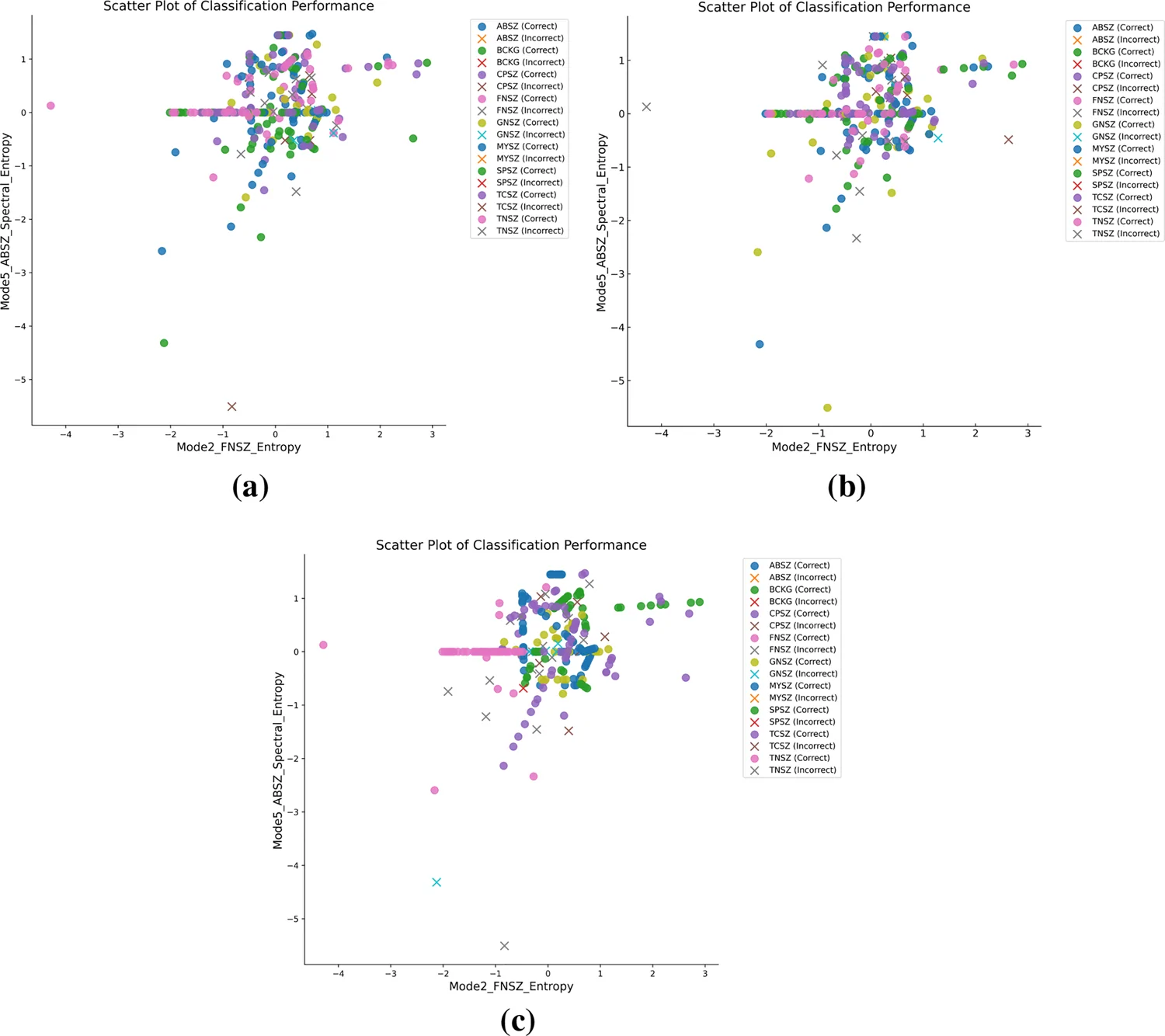
Scatter distribution of classifier accuracy across seizure classes (**a**) LightGBM + Logistic Regression (**b**) LightGBM + decision tree (**c**) XGBoost + Logistic Regression

**Fig. 19:**
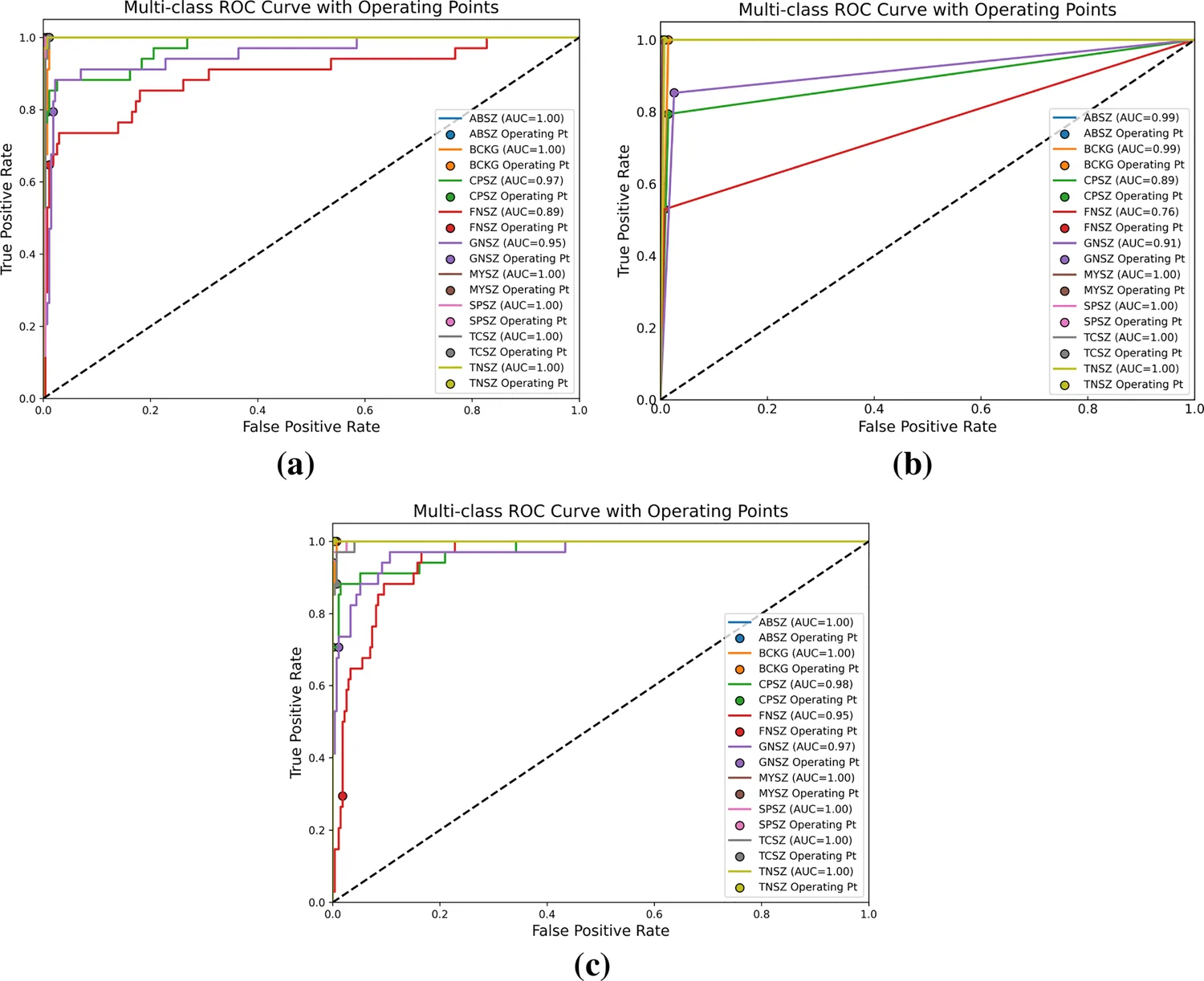
ROC performance curves demonstrating classifier convergence across classes (**a**) LightGBM + Logistic Regression (**b**) LightGBM + decision tree (**c**) XGBoost + Logistic Regression

Table 6 shows the hyperparameter settings of the suggested two-stage classifier models, LightGBM + Logistic Regression, LightGBM + Naive Bayes, LightGBM + Decision Tree, LightGBM + SVM, and XGBoost + Logistic Regression, and their baseline parameter values. Because EEG seizure classification contains very nonlinear and inseparable distributions in data, ensemble models like LightGBM and XGBoost are utilized to extract subtle feature interactions, while the secondary classifiers augment decision refinement. Rather than relying on a static 80:20 split, the evaluation was carried out with stratified K-fold cross-validation. To maintain the methodology’s rigor and prevent any information leak, all preprocessing steps related to class balancing have been performed separately for every training fold of cross-validation. Importantly, SMOTE has not been performed on the dataset before splitting it into training and testing parts. To confirm the stability of every model, numerous evaluation measures are used as described in Table 7 . The relative confusion matrices of classifiers, as presented in Fig. 16, indicate that the LightGBM + logistic regression model has the minimum misclassification compared to other pairings. In addition, the precision-misclassification trade-off is examined with Positive Predictive Value (PPV) and False Discovery Rate (FDR) presented in Fig. 17. Overall classification performance behaviors are depicted using scatter distributions in Fig. 18, and the ROC curves presented in Fig. 19 also prove the effectiveness and reliability of the proposed framework with regards to the reduced-channel scenario of EEG classification. Secondly, the hybrid learning model safeguards against weak patterns missed by the first-stage classifiers by ensuring that they are indeed caught by the second-stage learners, thus avoiding false predictions.

**Table 6:** Hyperparameter configuration of classifier models

Model	Model Settings	Assigned Values
LGBM + LR	Base learner	LightGBM (n_estimators = 100, random_state = 42)
	Meta learner	Logistic Regression (max_iter = 1000)
	Multi-class strategy	One-vs-Rest (OvR)
LGBM + NB	Base learner	LightGBM (n_estimators = 100, random_state = 42)
	Meta learner	Naïve Bayes
	Multi-class strategy	One-vs-Rest (OvR)
LGBM + DT	Base learner	LightGBM (n_estimators = 100, random_state = 42)
	Meta learner	Decision Tree
	Multi-class strategy	One-vs-Rest (OvR)
LGBM + SVM	Base learner	LightGBM (n_estimators = 100, random_state = 42)
	Meta learner	Support Vector Machine (RBF Kernel)
	Multi-class strategy	One-vs-One (OvO)
XGB + LR	Base learner	XGBoost (n_estimators = 100, random_state = 42)
	Meta learner	Logistic Regression (max_iter = 1000)
	Multi-class strategy	One-vs-Rest (OvR)

**Table 7:** Evaluation criteria for classifier performance

Metric	Mathematical Expression	Description
Accuracy (Acc)	$\mathrm{Acc} = \frac{TP + TN}{TP + TN + FP + FN}$	Overall correctness of the classifier.
Precision (Prec / PPV)	$\mathrm{Prec} = \frac{TP}{TP + FP}$	Correctness of positive predictions.
Specificity (Spec)	$\mathrm{Spec} = \frac{TN}{TN + FP}$	Ability to detect negative cases correctly.
Sensitivity (Sen / Recall)	$\mathrm{Sen} = \frac{TP}{TP + FN}$	Ability to detect positive (seizure) cases correctly.
Negative Predictive Value (NPV)	$\mathrm{NPV} = \frac{TN}{TN + FN}$	Reliability of negative predictions.
False Discovery Rate (FDR)	$\mathrm{FDR} = \frac{FP}{TP + FP}$	Proportion of false alarms among predicted positives.
Error Rate (Err)	$\mathrm{Err} = \frac{FP + FN}{TP + TN + FP + FN}$	Rate of incorrect classifications.
Kappa Statistics (Kap)	$\kappa = \frac{P_o - P_e}{1 - P_e}$	Agreement between prediction and truth beyond chance.
F1-Score (F1)	$\mathrm{F1} = \frac{2 \cdot \mathrm{Precision} \cdot \mathrm{Recall}}{\mathrm{Precision} + \mathrm{Recall}}$	Balance between precision and recall.

The staged decision process also alleviates variance and overfitting, which is a prevalent problem in high-dimensional EEG feature spaces. By combining complementary classifiers, the models tap both gradient boosting efficiency and statistical decision-making power, giving a more complete view of class boundaries.

**Fig. 20:**
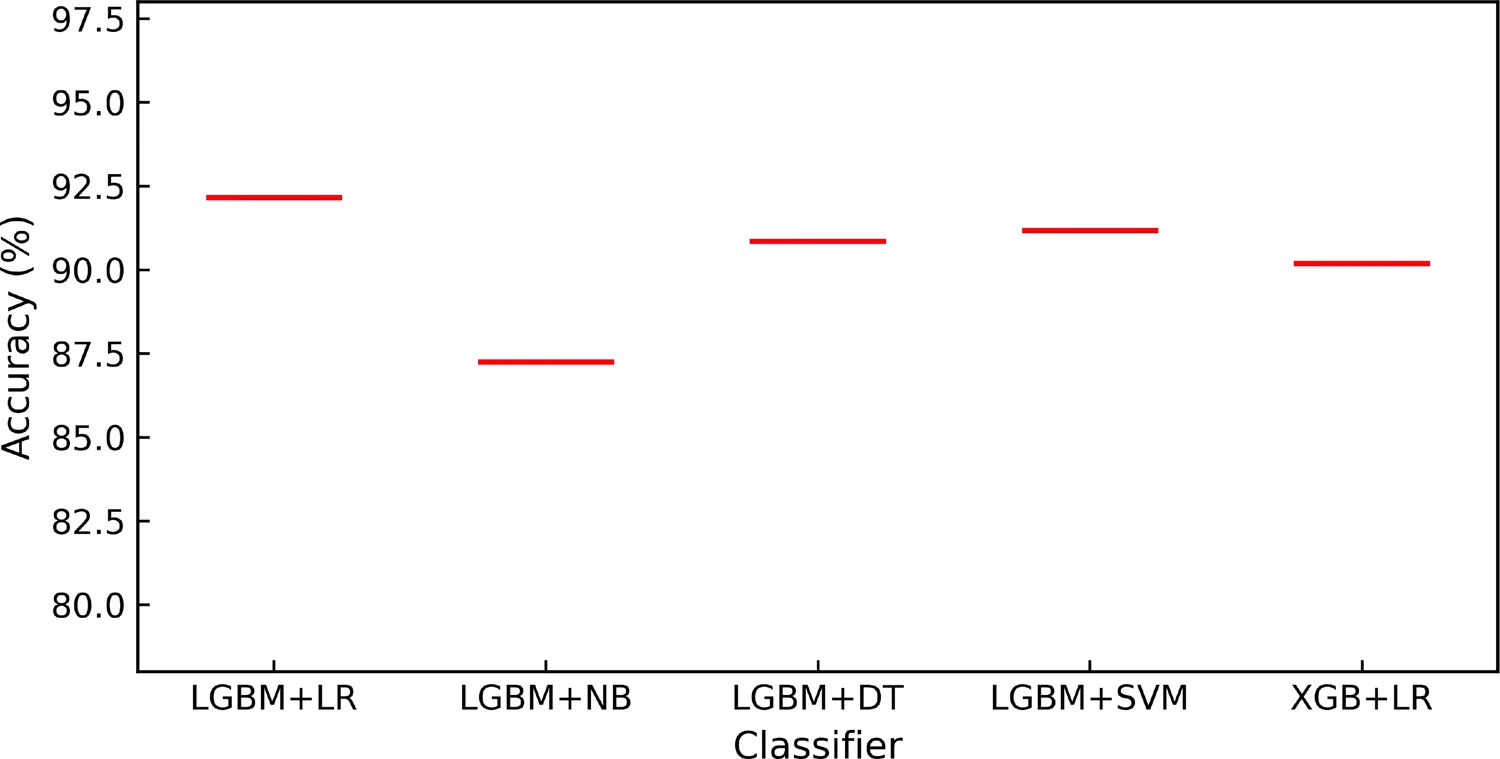
Box plot visualization of classifier accuracy distribution

To forecast the multiple seizure classes, the suggested two-stage classifiers were rigorously tested, and among those, the LightGBM + Logistic Regression model exhibited the most stable performance, with an overall classification accuracy of 92.16%, which is the best among all the tested models. Also, the weighted F1-score of 91.84%, and strong agreement characteristics are reflected by a Kappa statistic of 91.18%. The relative performance of all the classifiers in terms of F1-score, precision, sensitivity, and accuracy is shown in Table 8, emphasizing the better performance of the hybrid model compared to standalone models. Also, the statistical distribution of classification accuracy is shown in the form of a box plot in Fig. 20. As seen from the box plot, the LightGBM + Logistic Regression classifier shows the minimum variability between folds, as its generalization ability is stronger than those of its counterparts. Moreover, while hybrid model training complexity is slightly increased compared to single-stage learners, the higher predictive accuracy and robustness gains obviously compensate for the computational expense, rendering the proposed solution extremely well adapted to real-world multi-class seizure detection applications.

**Table 8:** Comparative evaluation of classifiers for multi-class seizure detection

Classifier	Class	%ACC	%PRE	%SEN	%SPE	%NPV	%PPV	%ERR	%FDR	F1-S	Kappa
LightGBM + Logistic Regression	ABSZ	99	94	100	99	100	94	1	6	0.97	0.97
	BCKG	99	92	100	99	100	92	1	8	0.96	0.95
	CPSZ	97	93	82	99	98	93	3	7	0.88	0.86
	FNSZ	94	81	65	98	96	81	6	19	0.72	0.69
	GNSZ	96	83	85	98	98	83	4	17	0.84	0.82
	MYSZ	100	100	100	100	100	100	0	0	1	1
	SPSZ	99	94	100	99	100	94	1	6	0.97	0.97
	TCSZ	99	92	100	99	100	92	1	8	0.96	0.95
	TNSZ	99	97	97	100	100	97	1	3	0.97	0.97
LightGBM + Decision Tree	ABSZ	99	89	100	99	100	89	1	11	0.94	0.94
	BCKG	99	89	100	99	100	89	1	11	0.94	0.94
	CPSZ	96	87	79	99	97	87	4	13	0.83	0.81
	FNSZ	94	90	53	99	94	90	6	10	0.67	0.64
	GNSZ	96	81	85	97	98	81	4	19	0.83	0.81
	MYSZ	100	97	100	100	100	97	0	3	0.99	0.98
	SPSZ	99	94	100	99	100	94	1	6	0.97	0.97
	TCSZ	99	94	100	99	100	94	1	6	0.97	0.97
	TNSZ	99	94	100	99	100	94	1	6	0.97	0.97
XGBoost + Logistic Regression	ABSZ	98	85	100	98	100	85	2	15	0.92	0.91
	BCKG	99	89	100	99	100	89	1	11	0.94	0.94
	CPSZ	97	91	85	99	98	91	3	9	0.88	0.86
	FNSZ	93	77	50	98	94	77	7	23	0.61	0.57
	GNSZ	96	80	82	97	98	80	4	20	0.81	0.79
	MYSZ	100	100	100	100	100	100	0	0	1	1
	SPSZ	100	100	97	100	100	100	0	0	0.99	0.98
	TCSZ	98	89	97	99	100	89	2	11	0.93	0.92
	TNSZ	100	97	100	100	100	97	0	3	0.99	0.98
LightGBM + SVM	ABSZ	100	89	100	99	100	89	0	11	0.94	0.94
	BCKG	100	89	100	99	100	89	0	11	0.94	0.94
	CPSZ	96	87	79	99	97	87	4	13	0.83	0.81
	FNSZ	94	90	56	99	95	90	6	10	0.69	0.66
	GNSZ	96	81	85	97	98	81	4	19	0.83	0.81
	MYSZ	100	97	100	100	100	97	0	3	0.99	0.98
	SPSZ	99	94	100	99	100	94	1	6	0.97	0.97
	TCSZ	99	92	100	99	100	92	1	8	0.96	0.95
	TNSZ	100	100	100	100	100	100	0	0	1	1
LightGBM + Naïve Bayes	ABSZ	98	87	97	98	100	87	2	13	0.92	0.91
	BCKG	98	87	100	98	100	87	2	13	0.93	0.92
	CPSZ	96	86	74	99	97	86	4	14	0.79	0.77
	FNSZ	92	65	50	97	94	65	8	35	0.57	0.52
	GNSZ	95	81	74	98	97	81	5	19	0.77	0.74
	MYSZ	100	97	100	100	100	97	0	3	0.99	0.98
	SPSZ	99	92	97	99	100	92	1	8	0.94	0.94
	TCSZ	98	89	94	99	99	89	2	11	0.91	0.90
	TNSZ	99	94	100	99	100	94	1	6	0.97	0.97

As part of assessing the strength and generalizability of the proposed framework, another set of experiments was done using the fixed data ratio of 40:40:20 for training, testing, and validation datasets, respectively. Specifically, in this experiment, 40% of the data sets were used to train the proposed algorithm, while the other 40% of the data sets served as testing samples. The rest of the data sets (20%) were then used to validate the proposed framework. As expected, an average classification accuracy of 83.87% was obtained using the proposed model and 40:40:20 training-testing-validation ratio. In addition, the precision and recall values were found to be 83.08% and 83.87% respectively, resulting in an F1-Score of 82.59%. The Kappa statistic was observed to be at 81.85% with the error rate at 16.13%.

### Mode-wise evaluation of VMD components

To explore further the contribution of individual decomposed Intrinsic Mode Function (IMFs) obtained by the VMD process, we have performed mode-wise analysis using the LightGBM + logistic regression model. The classifier performance per individual mode is tabulated in Table 9. The outcome indicates that Mode 3 yields the best accuracy of 97.66% with an F1-score of 0.8944, followed by Mode 2 at 97.11% accuracy. On the other hand, the lower frequency modes, like Mode 4, show relatively poorer performance, with only 90.44% accuracy and an F1-score of 0.5566. From the results shown above, it is evident that the crucial discriminating temporal and spectral features needed for seizure diagnosis are adequately captured by the selected VMD modes of the T3 and T4 electrodes. This analysis not only justifies the function of VMD in the separation of informative signal components but also accentuates the necessity of selectively exploiting the most informative modes for feature extraction to maximize overall classification efficiency.

**Table 9:** Performance of LightGBM + Logistic Regression across different modes

Classifier	Modes	%ACC	%PRE	%SEN	F1-S
LightGBM + Logistic Regression	Mode 1	96.66	82.66	84.33	0.8344
	Mode 2	97.11	87.55	87.66	0.8766
	Mode 3	97.66	89.22	89.77	0.8944
	Mode 4	90.44	55.22	56.66	0.5566
	Mode 5	96.66	83.22	84.55	0.8388

### Ablation-based evaluation of feature relevance in seizure detection

**Fig. 21:**
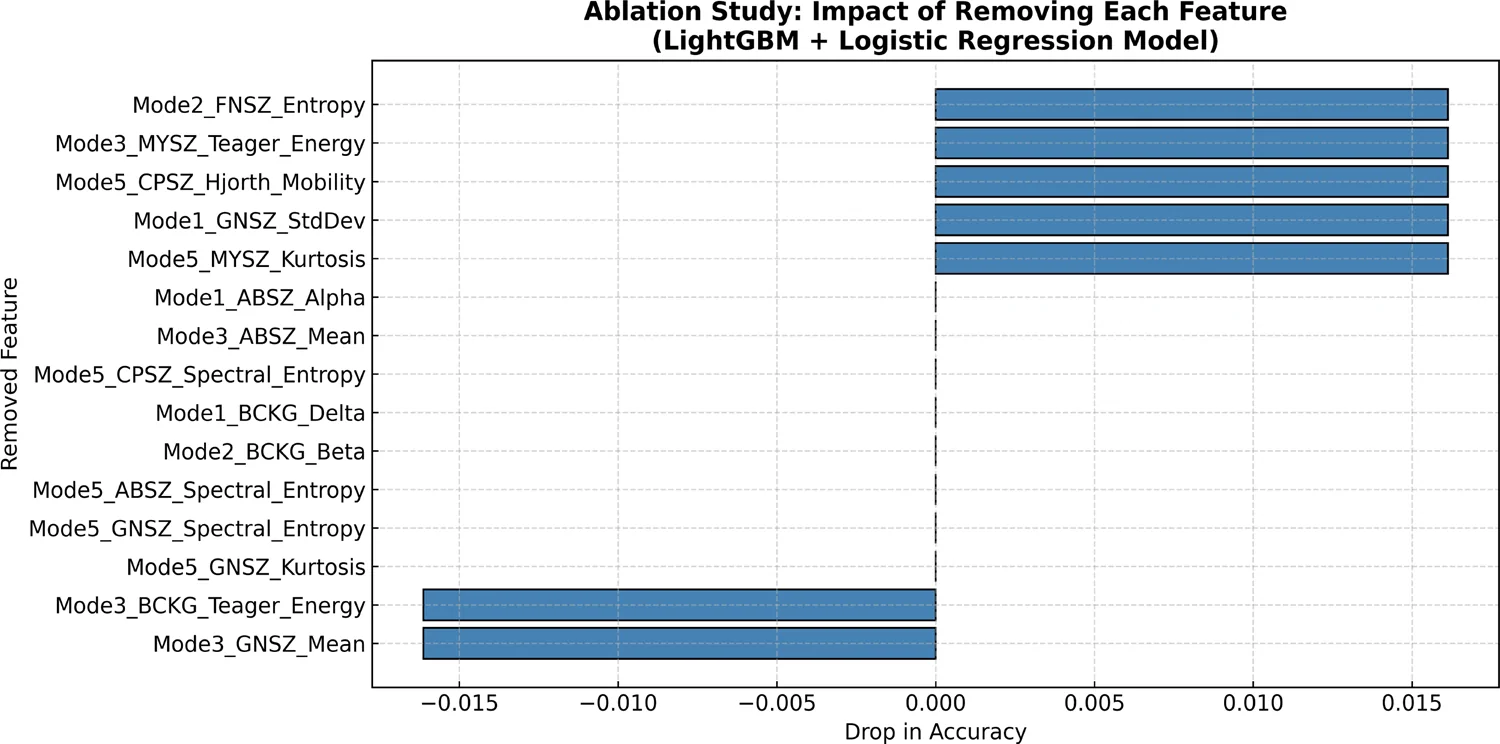
Ablation study: impact of removing each feature (LightGBM + Logistic Regression model)

To analyze the contribution of individual features and feature groups towards seizure classification, we performed a set of ablation experiments.We are performing this experiment on our base model (LightGBM).The outcome, as reflected in Tables 10 and 11, finds no hesitation in confirming that deleting individual features or entire feature groups causes a detectable drop in classification accuracy and F1-score. For example, removal of Mode3_MYSZ_Teager_Energy and Mode5_CPSZ_Teager_Energy brought down accuracy to 89.94% and F1-score to 0.0172, a decline by about 1.61% in accuracy (Table 10). Removal of Mode1_GNSZ_StdDev caused a similar deterioration, emphasizing the high discriminative ability of entropy and energy-related measures across various modes. The above observations are shown graphically in Fig. 21, presenting the sensitivity of classifier accuracy to removal of individual features.

**Table 10:** Ablation study: classifier performance after excluding single features

Feature No.	Feature Removed	% Accuracy	F1	%Accuracy Drop	F1 Drop
1	Mode_2_FNSZ_Entropy	90.33	0.8992	1.61	0.0174
15	Mode_3_MYSZ_Teager_Energy	90.33	0.8994	1.61	0.0172
8	Mode_5_CPSZ_Hjorth_Mobility	90.33	0.8994	1.61	0.0172
13	Mode_1_GNSZ_StdDev	90.33	0.8978	1.61	0.0192
14	Mode_5_MYSZ_Kurtosis	90.33	0.8978	1.61	0.0192
2	Mode_1_ABSZ_Alpha	91.94	0.9166	0	0
3	Mode_3_ABSZ_Mean	91.94	0.9166	0	0
9	Mode_5_CPSZ_Spectral_Entropy	91.94	0.9177	0	−0.0012
6	Mode_1_BCKG_Delta	91.94	0.9175	0	−0.0009
5	Mode_2_BCKG_Beta	91.94	0.9166	0	0

**Table 11:** Ablation study results for grouped feature exclusion

Feature Group	Feature Removed	% Accuracy	F1	% Accuracy Drop	F1 Drop
Group 4	Entropy Features	85.49	0.8435	6.45	0.0730
Group 2	Mode 2 Features	88.70	0.8779	3.22	0.0386
Group 3	Hjorth Features	90.33	0.8979	1.61	0.0186
Group 5	Statistical Features	91.94	0.9139	0	0.0026
Group 1	Mode 1 Features	95.17	0.9509	−0.0323	−0.0344

**Fig. 22:**
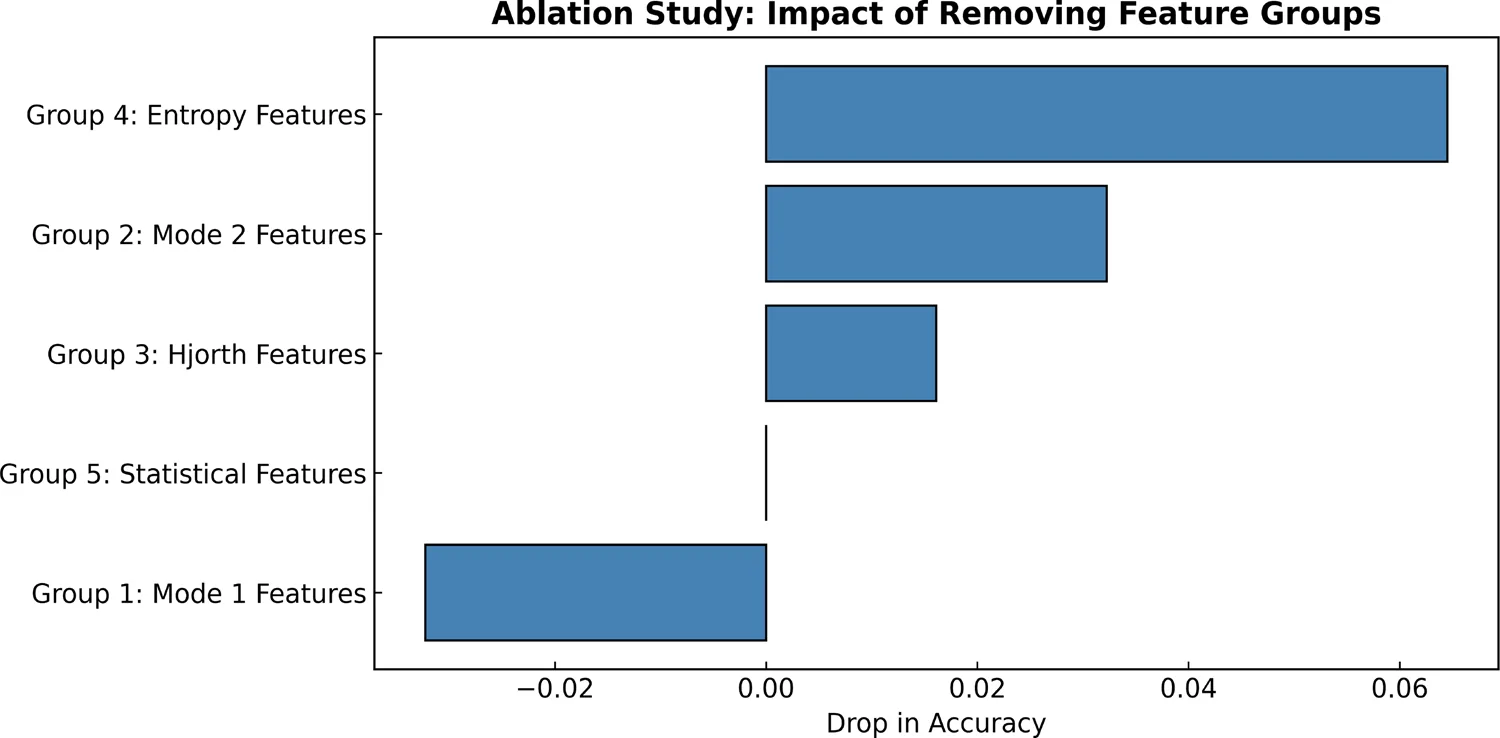
Ablation study: impact of removing feature groups

In the case of feature group exclusion, the effect was more notable. Specifically, the exclusion of all the entropy features (Group 4) gravely affected the classification accuracy, bringing it down to 85.94% and F1-score to 0.8435, which is a sharp decline of 6.45% in accuracy and 7.3% in F1-score (Table 11). In the same vein, removal of Mode 2 features (Group 2) lowered accuracy by 3.22%, again reinforcing the fact that entropy and mid-band frequency features are very critical in seizure class differentiation. Figure 22 illustrates the impact of feature group removal by domain.

It can be seen from the ablation study that the hybrid model not only exploits the complementary characteristics of time, frequency, and time-frequency features but also relies heavily on entropy-based metrics for reliable classification. Although the higher computational load introduced by the inclusion of all features initially may pose a limitation, the feature selection approach used afterwards guarantees that only the most significant features are kept, thereby improving generalization and computation as well.

### SMOTE on class imbalance

EEG seizure datasets, especially from TUSZ, tend to be severely class-imbalanced, with some seizure types having significantly fewer samples than others. This imbalance may result in skewed class distribution toward majority classes and can lead to weak minority class sensitivity. To mitigate this issue, the present work utilized the SMOTE, which creates artificial feature vectors for minority classes by interpolating among samples. This method effectively equalized the class distribution prior to training so that each type of seizure was represented equally. Consequently, the classifier attained better recall and balanced accuracy between classes and showed the importance of the role of SMOTE in achieving the generalization ability.

### LIME-based explainable AI analysis

**Fig. 23:**
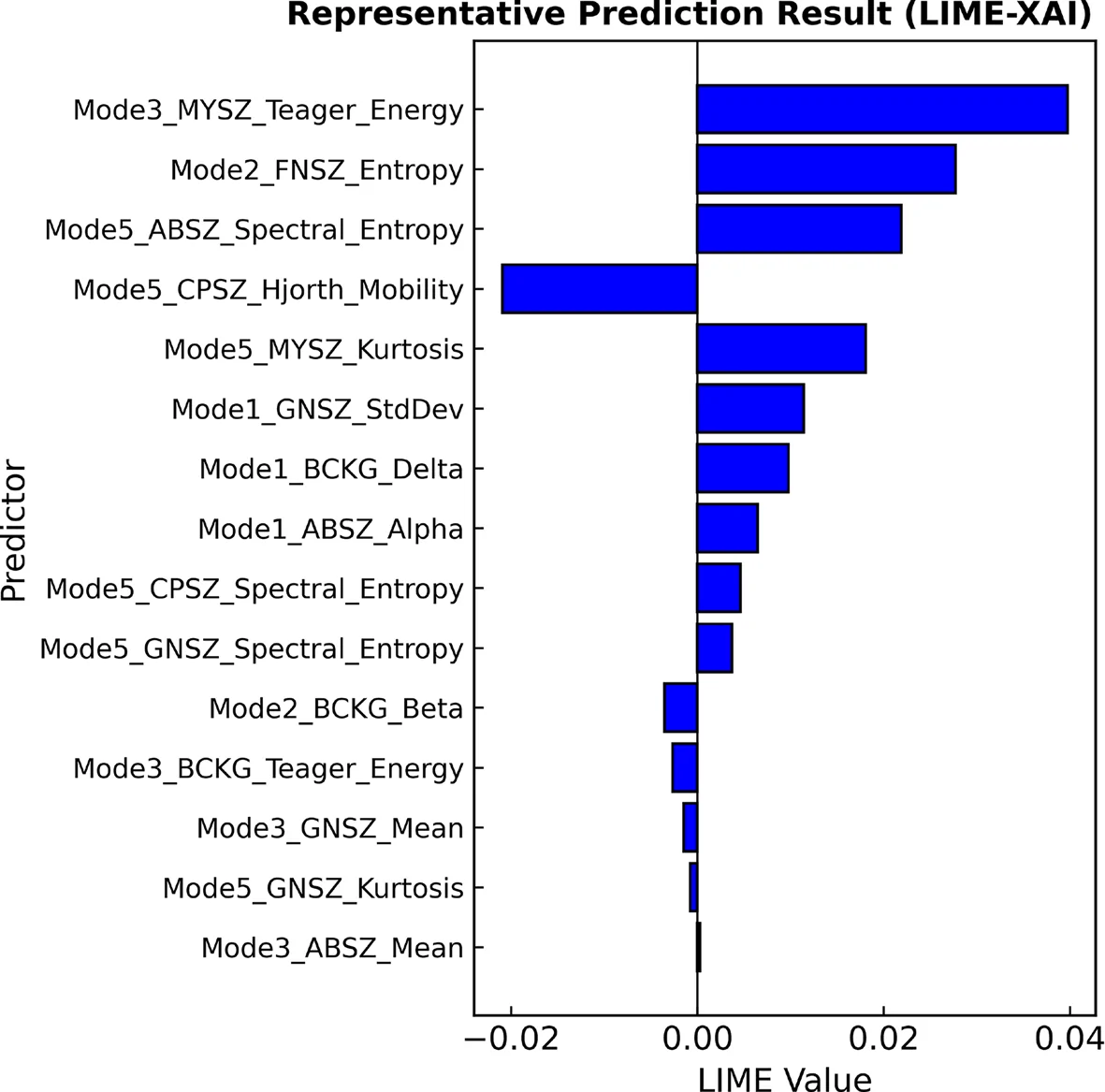
Representative prediction result (LIME-XAI)

To improve transparency for our proposed seizure classification framework, we utilized Local Interpretable Model-Agnostic Explanations (LIME) to examine each predictor’s contribution toward the predicted model decision. As depicted in (Fig. 23, iLIME explains the impact, both positive and negative, of the various predictors for a given EEG example. Predictors, including Mode3_MYSZ_Teager_Energy, Mode2_FNSZ_Entropy, and Mode5_ABSZ_Spectral_Entropy, exhibit much greater positive LIME values, implying each contributed or increased the classifier’s confidence towards this predicted seizure class. Other predictors, such as Mode3_ABSZ_Mean and Mode5_GNSZ_Kurtosis, displayed minor negative contributions, exhibiting the least opposing impact on the model classification. Predictors with larger positive LIME values, such as Mode5_CPSZ_Hjorth_Mobility and Mode5_MYSZ_Kurtosis, further demonstrate the discriminative ability of mobility, energy, and entropy-based descriptors derived from different VMD modes. This local explanation of the contribution of different predictors validates the relevance of these selected features while providing clinicians with interpretable and case-specific insights into the most important EEG features driving the model’s proposed decision. Consequently, the improves the interpretability and credibility of the XAI-integrated system proposed.

### Discussion

EEG signals are extremely non-stationary and complicated and offer essential information regarding the abnormal brain activity involved in seizures. However, visual interpretation of EEG traces is labor-intensive and prone to human errors, particularly for long-term monitoring data. To solve this problem, our research proposed an automatic machine learning–based seizure classifier framework utilizing Variational Mode Decomposition (VMD), feature engineering, and a two-stage stacked classifier. Particularly, the VMD method was utilized to separate the EEG signals into five modes, allowing both frequency band separation and denoising, and this revealed seizure oscillatory patterns related to them. As shown in Figs. 11, Figs. 12 and 2, the separated modes allowed for a more accurate spectral representation of seizure dynamics so that the process of feature extraction could be based on more informative neural elements. Time, frequency, and time-frequency domain features were all extracted from the five modes, after which redundancy was reduced with supervised hybrid feature selection techniques like SelectKBest MI-RFE and tree-based importance measures. This approach ensured that highly discriminative features only were retained for training.

Experimental results verify the efficacy of the designed LightGBM + Logistic Regression stacked model, which achieved a general classification accuracy of 92.16%, with excellent precision, recall, and F1 scores on seizure classes. In contrast to conventional methods that are based on a single domain of features, our multi-domain feature fusion enabled the model to learn various seizure features. The reduction in performance observed under the fixed partition evaluation further indicates that the revised leakage-free validation framework provides more realistic and unbiased performance estimation. In addition, using mode-wise analysis (Table 9), we noticed that lower-order modes like Mode 2 and Mode 3 showed higher classification accuracies (97.11% and 97.66%, respectively), while higher-order modes like Mode 4 demonstrated less discriminative power (90.44%). This confirms that seizure-specific neural oscillations are largely reflected in the early decomposition modes, and thus adaptive mode selection must be emphasized in follow-up studies.

In addition, the ablation studies (Tables 9 and 10; Figs. 21 and 22) validated the significance of entropy and Teager energy–based features in maintaining classification accuracy. The deletion of key entropy features resulted in accuracy declines of as much as 6.45%, reiterating their significance in seizure discrimination. The grouped feature ablation analysis highlighted that entropy features as a group constitute the most discriminative subset, although individual entropy features demonstrated minimal redundancy. The analysis offers greater interpretability of the role of every family of features in the classification.

To ensure the model’s interpretability and reliability, LIME-XAI was used to explain the individual contributions of features to a representative prediction (Fig. 23). The explanation displays strong, positive contributions of the following features: Mode3_MYSZ_Teager_Energy, Mode2_FNSZ_Entropy, and Mode5_ABSZ_Spectral_Entropy. This increased the model’s confidence in the prediction of the seizure class. In contrast, Mode3_ABSZ_Mean and Mode5_GNSZ_Kurtosis, had small, negative contributions. These local explanations augment the clinical trustworthiness of the system by explicitly documenting which EEG-derived predictors influence the model output prediction in each instance.

Comparison of the proposed framework to other recent state-of-the-art seizure classification techniques based on EEG is shown in Table 12. According to the results of the comparison, the proposed reduced-channel approach provided a competitive performance for multiclass seizure classification in the TUSZ database with 92.16% accuracy and F1-score 0.9184. In contrast to a number of other approaches using complex multichannel structures and large feature sets, the proposed one used only temporal EEG channels T3 and T4 along with hybrid MI-RFE feature selection and a stacked LightGBM-Logistic Regression model.

**Table 12:** Comparison of the proposed reduced-channel multi-seizure classification framework with recent studies

Reference & Year	Dataset	Features	Feature Selection	Classifier	%Acc	F1-S
Abbas et al. (2023) [25]	TUSZ	Nonlinear	Graph-based Feature Selection	RF	91.55	0.9155
Tuncer et al. (2022) [32]	TUSZ	Statistical	Correlation Method	SVM	90.30	0.9049
Zhao et al. (2024) [29]	TUSZ	ResNet Spatial + Temporal EEG Features	Automatic Deep Feature Learning	ResBiLSTM	91.27	0.9124
**Proposed Model**	**TUSZ**	**Time, Frequency & Time-Frequency**	**SelectKBest MI-RFE**	**LGBM + Logistic Regression**	**92.16**	**0.9184**

Last but not least, by solving the class imbalance issue with SMOTE, the model exhibited stability against imbalanced class distributions, guaranteeing equity between seizure types. In contrast to current methods in seizure classification that tend to underestimate imbalance management or limit themselves to sole-domain features, our research provides three major contributions:

- Adaptive signal decomposition via VMD for all modes
- Multi-domain feature fusion and selection to extract only relevant features, and
- two-stage stacked classification with LightGBM and logistic regression for better Generalization.

In general, this paper shows that the suggested framework can be an accurate and interpretable multi-class seizure detection tool. With optimization and edge device deployment (e.g., NVIDIA Jetson or Cortex-M platforms), the framework can be potentially used for real-time clinical seizure monitoring and decision support.

### Limitations of the proposed framework

Despite the proposed seizure classification framework achieving an impressive overall accuracy of 92.16% accuracy, some limitations that we need to note are as follows. First, we should mention that VMD-based decomposition and multi-domain feature extraction are not without computational burden for harmful real-time deployment on resource-constrained platforms, despite the improvement in discriminative power. Second, to address the class imbalance we encountered, we applied a SMOTE-based resampling method that created synthetic samples; however, these samples likely do not fully account for the complex physiological variations of minority seizure types and may therefore impact robustness in real-world applications. Despite yielding promising results in terms of classification accuracy, there are several limitations associated with the current study. First, the framework implemented by us involves only two temporal EEG channels (T3 and T4). As a result, the full extent to which generalized seizures propagate spatially is not accounted for. Channel reduction increases efficiency and allows for wearable EEG monitoring, but adding more electrodes to the montage could further improve results.

While the proposed framework showed encouraging results on the TUSZ dataset, the current work is limited to just one large-scale seizure dataset. Conducting experiments using the proposed technique on other EEG datasets such as the CHB-MIT dataset would be a good means of evaluating the generality of the method as well as validating its dataset independence. There are several challenges associated with testing a generalized seizure classification scheme across EEG datasets owing to differences in electrode placement, seizure annotation schemes, sampling rates, and demographics of patients, among others. The current work thus considers cross-dataset testing and multichannel benchmarking as potential avenues for future investigation.

While this paper uses the stratified K-fold cross-validation methodology instead of subject-independent evaluation techniques such as the LOSO technique, the validation scheme used in the research provides a robust performance assessment. However, patient-independent generalization capability of the method can be assessed by employing the LOSO validation technique in a more realistic setting. Thus, it is worth investigating the real-world application ability of subject-independent seizure classification via the LOSO validation method. In this paper, although efficiency in computations was achieved by employing fewer channels of the EEG signal along with lightweight machine learning methods, no real-time performance analysis was provided. Thus, it can be considered a key area of future investigation.

Moreover, we should note that we used the TUSZ dataset for our study, which is certainly comprehensive, but data was collected in a controlled setting; we have not evaluated performance in the presence of noise and heterogeneous clinical environments. Finally, we utilized the T3 and T4 temporal channels exclusively for the proposed framework, which suggests further exploration of the generalizability of the results across different electrode montages or higher-density EEG systems is warranted.

## Conclusion

In this study, a reduced-channel EEG-based multi-seizure classification approach was developed based on the TUSZ dataset. Specifically, the methodology involved VMD, multi-domain feature extraction, MI-RFE, and stacked LightGBM-logistic regression classifiers for automated multi-seizure classification using only the T3 and T4 temporal EEG channels. To eliminate potential bias in the experimental results, fold-wise SMOTE was performed only in the training folds using stratified 10-fold cross-validation. The experimental results showed promising multi-seizure classification performance with high overall accuracy, weighted F1-score, and robust multi-class ROC-AUC properties among nine seizure classes. In addition, the results of another experiment performed with a fixed 40:40:20 training, testing, and validation split further validated the robustness and generalization ability of the proposed methodology under hold-out validation settings. The achieved results of classification accuracy 92.16% suggest that reduced-channel EEG signals are capable of retaining discriminative seizure information while providing computational efficiency for building low-complexity and wearable seizure monitoring devices.

### Advantages of the proposed framework

The proposed framework offers several important advantages for reduced-channel EEG-based seizure classification:

- The use of only T3 and T4 temporal EEG channels reduces computational complexity and supports lightweight seizure monitoring applications.
- VMD-based decomposition effectively captures seizure-related temporal and spectral characteristics from nonstationary EEG signals.
- The hybrid MI-RFE feature selection strategy reduces feature redundancy while preserving discriminative seizure-related information.
- The stacked LightGBM–Logistic Regression classifier improves classification robustness through ensemble learning.

### Future research directions

Future work will focus on validating the proposed framework by using other multichannel EEG data sets, such as the CHB-MIT data set, and analysing the generalisability of the system across different data sets, as well as evaluating the inference time, memory requirements, and real-time capabilities of the framework for EEG monitoring devices. The additional inclusion of scalp electrode sensors, along with multimodal EEG representation, may lead to better localisation and classification of seizures. The future research direction will involve the study of patient-independent learning, real-time seizure detection methods, feature fusion, and wearable EEG system implementations.

## Data Availability

No datasets were generated or analysed during the current study.
